# Cytomegalovirus infection during pregnancy: cross-sectional survey of knowledge and prevention practices of healthcare professionals in French-speaking Switzerland

**DOI:** 10.1186/s12985-024-02318-w

**Published:** 2024-02-21

**Authors:** Pauline Sartori, David Baud, Begoña Martinez de Tejada, Alexandre Farin, Marie-Claude Rossier, Wawrzyniec Rieder, Sylvie Rouiller, Romaine Robyr, Gaston Grant, Béatrice Eggel, Adrien Bruno, Maeva Pretalli, Bertrand Gainon, Romina Capoccia-Brugger, Laetitia Ammon-Chansel, Clarisse De Courten, Nathalie Beurret-Lepori, Jonathan Baert, Yvan Vial, Léo Pomar

**Affiliations:** 1 School of Health Sciences (HESAV), HES-SO University of Applied Sciences and Arts Western Switzerland, Avenue de Beaumont 21, 1011 Lausanne, Switzerland; 2 Department Woman-Mother-Child, Lausanne University Hospital and University of Lausanne, 1011 Lausanne, Switzerland; 3 Obstetrics Division, Department of Pediatrics, Gynecology and Obstetrics, Faculty of Medicine, University Hospitals of Geneva, 1205 Geneva, Switzerland; 4 Obstetrics Unit, Hospital Riviera-Chablais, 1847 Rennaz, Switzerland; 5 Dianecho, 1205 Geneva, Switzerland; 6 Service of Gynecology and Obstetrics, Ensemble Hospitalier de La Côte, 1110 Morges, Switzerland; 7 Echofemme, 1204 Geneva, Switzerland; 8 Department of Gynecology and Obstetrics, Cantonal Hospital, 1752 Villars-Sur-Glâne, Switzerland; 9 Maternal-Fetal Medicine, Point-f Women’s Medical Centre, 1700 Fribourg, Switzerland; 10 Maternity Ward, Hôpital du Jura, 2800 Delémont, Switzerland; 11 Department of Obstetrics and Gynecology, Réseau Hospitalier Neuchâtelois, 2000 Neuchâtel, Switzerland; 12 Geneva section of the Swiss Federation of Midwives, 1248 Hermance, Switzerland; 13 Department of Gynaecology and Obstetrics, Regional Hospital EHNV, 1400 Yverdon, Switzerland; 14 Imagerie du Flon, Lausanne, Switzerland

**Keywords:** Cytomegalovirus, CMV, Pregnancy, Healthcare, Knowledge, Awareness, Screening, Prevention, Practices, Recommendations

## Abstract

**Background:**

Lack of Cytomegalovirus (CMV) knowledge among healthcare professionals has been proven to be the main threat to pregnant women's awareness, preventing them from reducing the risk of infection. The aims of this study were to assess the knowledge and practices of French-speaking Swiss perinatal professionals in terms of CMV prevention, as well as the sociodemographic-professional factors that influence them.

**Methods:**

This observational study used a cross-sectional design to collect data-via an anonymous electronic questionnaire in French distributed to gynecologists-obstetricians, general practitioners and midwives via various channels: e-mails and social networks of partner centers, professional associations, and conferences. The 41-item questionnaire collected data on sociodemographic and professional characteristics, general CMV knowledge, national recommendation knowledge and prevention practices. Univariable and multivariable analyses were performed.

**Results:**

A total of 110 gynecologist-obstetricians, 5 general practitioners and 226 midwives participated in the study. While more than 80% of practitioners were familiar with protective hygiene measures, significant gaps were highlighted concerning the transmission routes, as well as the signs of short- and long-term congenital CMV infection. Regarding practice, 63.3% of participants provided information on CMV to their patients, mainly during the first antenatal visit. Among those who did not, lack of knowledge and forgetfulness were the two main reasons cited. Concerning systematic screening, 45.7% of participants offered it to their patients, and 37.3% only offered it to “at risk” groups. The existence of national guidelines on CMV was known by 62.0% of participants. Multivariable analysis revealed that working as a gynecologist-obstetrician was independently associated with higher score of preventive practices, while performing ultrasound or preconception consultations was independently associated with a higher score of general CMV knowledge, and working in a university hospital was independently associated with a higher score of Swiss recommendation knowledge. A level of training higher than the basic medical or midwifery diploma and participation in fetal medicine symposia both promote a higher score of CMV knowledge and prevention practices in line with current recommendations.

**Conclusion:**

This study confirms the significant gaps in CMV knowledge among French-speaking Swiss caregivers along with the heterogeneity of their prevention practices. To raise awareness among pregnant women and reduce the burden of congenital CMV infections, improving professional knowledge through access to specific training and standardizing practices should be a national priority.

**Supplementary Information:**

The online version contains supplementary material available at 10.1186/s12985-024-02318-w.

## Introduction

Cytomegalovirus (CMV) is a DNA herpes virus within the family of herpesviruses (HHV-5: human herpesvirus 5) with an estimated seroprevalence of 50% in Switzerland. CMV is the leading cause of teratogenic congenital infections [1–5], affecting 2.3% of pregnant women each year and 0.5–1% of live births [6–9]. Maternal primary infections are caused by direct or indirect exposure to infected body fluids (saliva, urine, blood or sexual secretions) [10]. Reinfections and reactivations of endogenous strains can result in nonprimary infections [11, 12]. In immunocompetent pregnant women, most CMV infections are asymptomatic, but 10–20% may experience flu-like symptoms [13]. In Switzerland, the rate of primary CMV infection during pregnancy is estimated at 0.5–1%, affecting 400–800 pregnant women each year [14]. Most infected neonates are asymptomatic, but 10–15% will present signs of infection, such as microcephaly, low weight and seizures at birth [9]. Up to 15% of asymptomatic neonates will develop late-onset neurosensory disorders. Symptomatic neonates have a significantly worse prognosis with a 40–60% risk of permanent sequelae, such as sensorineural hearing loss (SNHL), cognitive impairment, retinitis, and cerebral palsy [9, 15, 16]. Congenital CMV (cCMV) infections are recognized as the leading cause of nongenetic neurosensory impairment [3–5] and are responsible for 10% of all cases of cerebral palsy in children [17]. In Switzerland in 2021, 447 children were born with CMV infection, including 45 newborns with symptomatic infection at birth [14]. The rate of termination of pregnancy due to CMV congenital infection is unknown.

In the absence of an available active vaccine, the application of preventive hygiene measures by pregnant women has proven to be the most effective way of avoiding contamination [18–21]. Although systematic screening for CMV is still controversial [14, 22], there is scientific consensus on providing information on the virus and reducing the exposure of pregnant patients preconceptionally or as early as possible during pregnancy [14]. Rigorous application of hygiene measures could reduce seroconversion rates by as much as 50% [19], making it all the more important for pregnant women to be aware of specific hygiene practices [10, 23]. Despite international consensus on the importance of preventive information to reduce the risk of infection in pregnant women, an important number of studies have shown significant gaps in CMV awareness in the pregnant woman population [24, 25] and correlated preventive behavior with the awareness of the virus received from healthcare professionals [26, 27]. In Switzerland, the Swiss Society of Gynecology and Obstetrics (SSGO) recommends that “all pregnant women or women considering pregnancy should be informed as soon as possible about the risks of CMV infection and recommended preventive hygiene measures to reduce this risk” [14]. However, a national study investigating the awareness of CMV among pregnant women in 2015 showed that only 39% of participants had heard of CMV before delivery, and only 20% had received information about preventive measures during pregnancy [28]. Preliminary results from a study by Pomar et al*.* (2022) of 834 pregnant women in the French-speaking part of Switzerland show that little has changed over a decade, as more than a third of participants were unaware of CMV and ignored whether or not they had been screened for CMV during pregnancy [29]. Lack of knowledge regarding the modes of virus transmission [30–32], of potential maternal and fetal symptoms [23, 30, 31, 33], of therapy options [23, 30, 31, 33] and the lack of clear national or international guidelines to guide their clinical practice [23, 34] were identified as the main factors interfering with an effective preventive message by the clinician.

Consequently, investigating and monitoring the national level of professional knowledge about CMV, as well as professional practices specific to the virus and their knowledge of national recommendations, seemed relevant from the perspective of reducing the number of cCMV infections in Switzerland.

The main objectives of this study were to investigate CMV knowledge (including national recommendation) and prevention practices of healthcare professionals directly involved in pregnant women’s care. The secondary objective was to determine the correlation between the participants’ knowledge and practices and their sociodemographic and professional characteristics.

## Method and material

### Study design, data collection and population of interest

This observational study used a cross-sectional design to collect data on variables of interest (knowledge and prevention practices related to CMV) and the characteristics of participants in a single survey. Given that Switzerland is a country divided into three regions, with German, French and Italian as the respective languages, it was determined that only healthcare professionals practicing in the French-speaking part of the country would be included in this study. Therefore, this survey, conducted by the School of Health Sciences (HESAV) in partnership with hospitals and private practices in French-speaking Switzerland, took place in western Switzerland between October 2022 and May 2023. Data were collected via a 41-question anonymous questionnaire on healthcare professionals' knowledge and attitudes toward CMV (Additional file 1). Physicians and midwives were able to access the questionnaire in different ways, including the e-mail lists of partner centers and various medical (SSGO, Swiss medical federation) and midwifery (Swiss federation of midwives) associations, social media of Swiss hospitals, personal professional contacts, website from the Groupement Romand of the Swiss Society of Gynecology and Obstetrics (GRSSGO) and posters presented at four medical congresses. All physicians and midwives practising in French-speaking Switzerland and in contact with women in the periconceptional or antenatal period were invited to complete the questionnaire. The target population for the region of interest included 507 gynecologist-obstetricians, 1570 independent and hospital midwives, and 16 general practitioners providing pregnancy follow-up care [35–37]. The target population thus comprised 2093 practitioners eligible to take part in the study. Finally, an IP (Internet Protocol) tracker was implemented to prevent multiple responses from participants.

### Data sources/measurement

The questionnaire was written in French, based on a previously validated model in English and French [30, 31, 38] to which we added variables related to knowledge, practice attitude of the latest recommendations on CMV [14] and antenatal treatment of CMV. The added items were validated by an expert committee, the internal consistency was tested in a pilot study using Cronbach’s alpha for each domain (> 0.7) among 30 participants, and the reproducibility of the whole questionnaire was validated by a retest procedure at one-week intervals in the same group of participants (validation of the reproducibility of answers for a kappa index > 0.8). The questionnaire included questions on CMV knowledge (transmission route, maternal and fetal symptoms, preventive measures), on professionals’ attitudes toward patients (prevention information and screening) and on knowledge and adherence to national recommendations. The proposed answers were single or multiple answers, and some of them proposed false answers (i.e. false fetal symptoms). To compare the baseline characteristics of healthcare professionals with good knowledge of CMV with those with poor knowledge of CMV, a sample size of 300 to be recruited was initially based on a binary dependent variable (score ≥ 15 versus score < 15) to reach a delta of 0.05 for five independent variables, with a power of 0.8 and a significance level of 0.05 in multivariable linear regressions. This sample size could also permit to use continuous values for dependent variables.

### Variables

Collected information addressed basic characteristic variables such as profession (obstetrician-gynecologist, general practitioner, midwife), place of work (university or regional hospital, clinic, private practice, birthing center), years of professional experience, field of practice (preconceptional, antenatal or postnatal consultation, prenatal diagnosis, outpatient care, labor ward, other), participation in fetal medicine symposia or recent training on CMV (in the last two weeks, either a dedicated conference or a congress where one of the topics was CMV). Information related to professional knowledge of CMV was collected through variable addressing transmission route, clinical potential signs and symptoms in a pregnant woman and fetus/newborn, possible long-term effects of cCMV infection, severity of fetal infection depending on the term, possibility of congenital infection after reactivation or reinfection, possibility of in utero treatment, and knowledge of hygiene measures to protect against CMV. Professional practice attitude information was assessed thanks to variables addressing prevention information given to pregnant women, the timing at which information is given, the reasons why information is not given, if screening is proposed, the timing at which screening is proposed, and the target population for screening. Knowledge of SSGO recommendations for screening and prevention of CMV in pregnancy was assessed through variables addressing knowledge of recommendations on prevention, screening and management elaborated by expert committees. A final variable named “recent training” was created to assess whether the participant had responded to this study within two weeks of receiving specific CMV training.

### Outcomes

To achieve the main objective, a primary outcome was defined, aimed at assessing the general knowledge of the professionals. An overall knowledge score of up to 42 points was generated, with 1 point awarded for each correct answer. Two secondary outcomes were then developed to assess professionals' practice attitudes, focusing on prevention and screening, as well as their knowledge and adherence to the national recommendations drawn up by the SSGO. Clinical practices were assessed using a four-point score. One point was awarded for the following variables: information provided, information given at first consultation, systematic screening proposed, and discussed during the first consultation. For practitioners who do not offer systematic screening, one point was awarded for responses relating to targeted screening for “ultrasound signs” and “patients in contact with young children considered” as high-risk patients. Knowledge and adherence to the recommendations were assessed using a seven-point score, in which one point was awarded for each correct response regarding current national recommendations.

### Statistical analysis

Quantitative variables were analyzed according to their distribution. The normality of their distribution was estimated using the skewness and kurtosis test. The means and standard deviations of variables with a normal distribution are presented, as well as the medians and interquartile ranges of variables presenting a nonnormal distribution. Univariate analyses were carried out to identify the sociodemographic and professional characteristics potentially associated with the primary outcome "knowledge of CMV" and with the secondary outcomes "professional practice" and "knowledge of recommendations". Analysis with binary or categorical variables included chi-square and Fisher’s exact tests. Univariate analyses of quantitative dependent variables were carried out using Student’s t or Mann‒Whitney U tests, depending on their distribution. Multivariable analyses were carried out using logistic or general linear regression to identify potential factors independently associated with the different outcomes. The multicollinearity of the variables was checked using correlation tests, and if two covariates were correlated with a coefficient > 0.70, one of these variables was removed from the multivariable models. The results are presented in adjusted coefficient (*a*Coeff) presenting the difference in the predicted value of the response variable for each one-unit change in the predictor variable, supposing that all other predictors are held constant. Only variables with a *p* value < 0.10 in the univariate model were included in the multivariable models. A significance level of 0.05 was set for interpretation of the multivariable analyses. Statistical analyses were performed using Stata 16 (StataCorp, https://www.stata.com).

### Missing data

A complete case analysis rather than multiple imputations was used in this study due to the presumed low level of missing data (< 5% per variable).

## Results

### Respondent sample

**Fig. 1:**
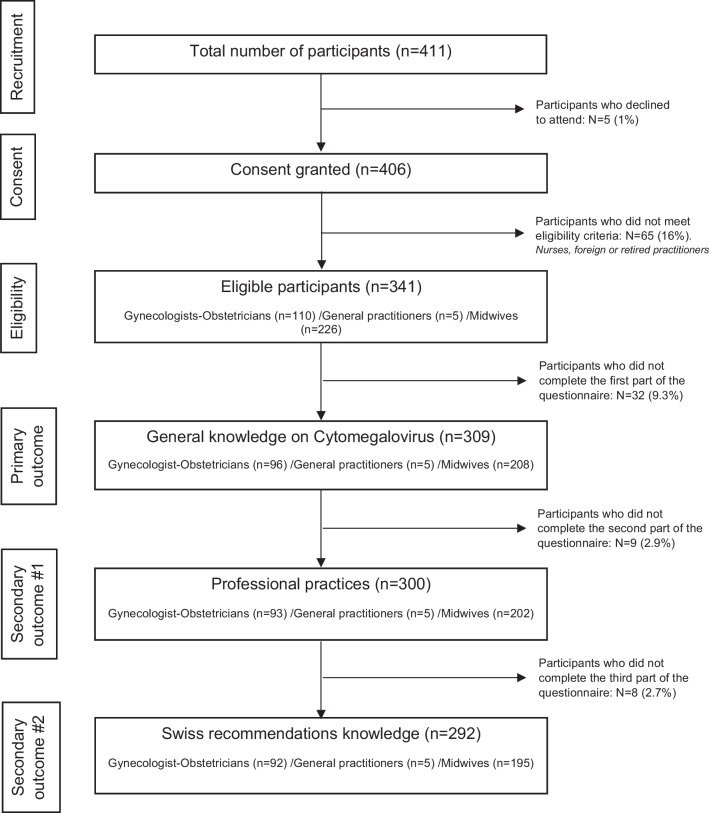
Flow chart

A total of 406 practitioners agreed to participate in the online study. Sixty-five of them (16%) did not meet the eligibility criteria (nurses, foreign or retired practitioners, for example). As a result, a total of 341 practitioners met features that enabled further participation, including 110 gynecologist-obstetricians, 5 general practitioners and 226 midwives, which represents 16.2% of the 2093 eligible practitioners. Of these participants, 96 gynecologists-obstetricians, 5 general practitioners and 208 midwives answered questions relating to the primary outcome of this study (n = 309, 76.1% of participants). Concerning the secondary outcomes of this study, 300 (97.1%) and 292 (94.5%) participants completed the items used to generate the scores related to professional practice and to knowledge of Swiss recommendations (Fig. 1). The median time required to complete the questionnaire was 8 min (IQR 5–11).

### Baseline characteristics

The basic and professional characteristics of the participants are shown in Table 1. Midwives represented the largest group included (67.3%; 208/309), followed by gynecologists-obstetricians (31.1%; 96/309) and general practitioners (1.6%; 5/309). A majority of participants (52.8%; 163/309) held a level of training higher than the basic training needed for their profession (i.e., BSc for midwives and Federal diploma in medicine for physicians) and had more than 10 years of practice (62.5%; 193/309). The main places of practice reported were private practice (38.2%; 118/309), university hospitals (37.5%; 116/309), and regional hospitals (27.2%; 84/309). The practices most frequently carried out by the participants were postpartum follow-up (67%; 207/309), pregnancy follow-up (61.2%; 189/309) and ultrasonography (54.4%; 168/309). Only 34% of participants reported attending multidisciplinary fetal medicine symposia (105/309), and 26.5% (82/309) responded to this survey within two weeks of CMV-specific training.

**Table 1:** Baseline characteristics of participants

Baseline characteristics	Gynecologists-obstetricians	General practitioners	Midwives	Total
	*n* = 96	*n* = 5	*n* = 208	*n* = 309
	*n* (%)	*n* (%)	*n* (%)	*n* (%)
Highest level of training				
Bachelor's degree	–	–	127 (61.1)	127 (41.1)
CAS or university diploma of specialization	5 (5.2)	–	42 (20.2)	47 (15.2)
Master's degree	1 (1.04)	–		34 (11.0)
Federal diploma in human medicine	16 (16.7)	2 (40)	33 (15.9)	18 (5.8)
Specialist title	48 (50)	3 (60)		51 (16.5)
Specialist title with advanced training in maternal–fetal medicine	12 (12.5)	–	–	12 (3.9)
Doctorate (MD and / or PhD)	14 (14.6)	–	5 (2.4)	19 (6.2)
Professional age after diploma				
< 1	2 (2.1)	1 (20)	8 (3.9)	11 (3.6)
1–5	14 (14.6)	–	29 (13.9)	43 (13.9)
6–10	14 (14.6)	–	48 (23.1)	62 (20.1)
> 10	66 (68.8)	4 (80)	123 (59.1)	193 (62.5)
Practice location (multiple choices)				
Private practice	54 (56.3)	4 (80)	60 (28.9)	118 (38.2)
University Hospital Centre	24 (25)	1 (20)	91 (43.8)	116 (37.5)
Peripheral hospitals	31 (32.3)	–	53 (25.5)	84 (27.2)
Private hospital	8 (8.3)	–	3 (1.4)	11 (3.6)
Birth center	–	–	12 (5.8)	12 (3.9)
Consultation type (multiple choices)				
Pregnancy follow-up	83 (86.5)	4 (80)	102 (49)	189 (61.2)
Emergency ObGyn consultations	80 (83.3)	3 (60)	77 (37)	160 (51.8)
Preconception consultations	57 (59.4)	4 (80)	9 (4.3)	70 (22.7)
Routine fetal ultrasound	55 (57.3)	–	18 (8.7)	73 (23.6)
Oriented fetal ultrasound	63 (65.6)	–	12 (5.8)	75 (24.3)
Expert fetal ultrasound	15 (15.6)	–	5 (2.4)	20 (6.5)
Birth preparation	13 (13.5)	–	91 (43.8)	104 (33.7)
Post partum	62 (64.6)	–	145 (69.7)	207 (67)
Childbirth	67 (69.8)	–	103 (49.5)	170 (55)
Attend multidisciplinary fetal medicine symposia	62 (64.6)	–	43 (20.7)	105 (34)
Recent training	32 (33.3)	3 (60)	47 (22.6)	82 (26.5)

### Participants' knowledge of CMV

**Fig. 2:**
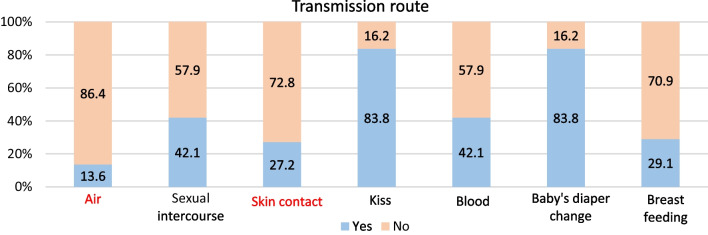
Participants' knowledge of the virus transmission route. *Note* Incorrect answers are shown in red

Of the seven transmission routes proposed, five were correct, two of which were correctly identified by more than half of the participants: kissing on the mouth and changing the baby's diapers (83.8%, 259/309 for both). Approximately one in 10 participants misidentified air as a possible route of virus transmission (13%; 42/309), and a quarter (27.2%; 84/309) wrongly identified skin contact as a possible route of virus transmission. Breastfeeding is the correct transmission route least known to the panel of respondents (29.1%; 90/309; Fig. 2).

With regard to maternal symptoms, the majority of respondents were able to identify the three correct answers out of a total of seven (asymptomatic 86.4%; 267/309, fever 64.7%; 200/309, flu-like syndrome 83.5%; 258/309).

**Fig. 3:**
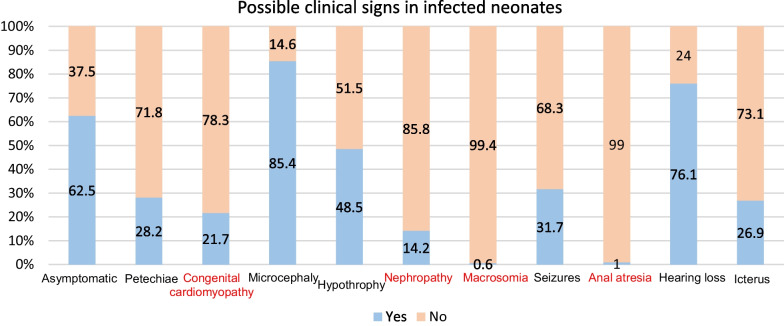
Knowledge of possible clinical signs in CMV-infected newborns. *Note* Incorrect answers are shown in red

Concerning potential neonatal clinical symptoms, most of the participants correctly identified microcephaly (85.4%; 264/309) and hearing loss (76.1%; 235/309) as potential clinical signs of cCMV infection. Fewer than half of the participants identified other correct signs of cCMV infection, such as petechiae (28.2%), asymptomatic (37.5%), hypotrophy (48.4%), seizures (31.7%) and icterus (26.9%) (Fig. 3). Less than a third of participants misidentified potential symptoms, and one in five participants also misidentified congenital cardiomyopathy as a potential neonatal symptom (21.7%; 67/309).

**Fig. 4:**
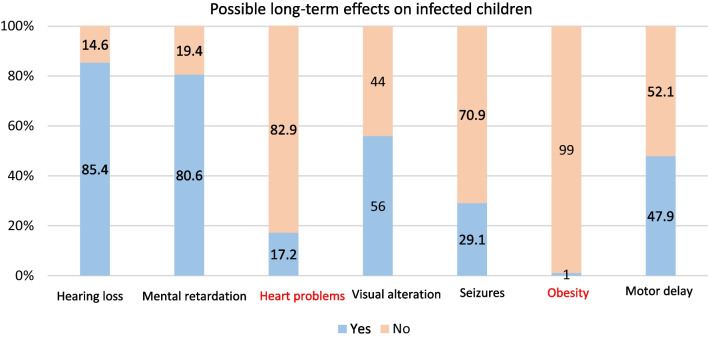
Knowledge of possible long-term effects on infected newborn. *Note* Incorrect answers are shown in red

In terms of potential long-term sequelae of cCMV infection, the most frequently identified were hearing loss (85.4%; 264/309) and mental retardation (80.6%; 249/309). The risk of seizures (29.1%; 90/309) was the sign least recognized by participants. Over four-fifths of the panel correctly identified heart problems (17.2%; 53/309) and obesity (1%; 3/309) as false symptoms of cCMV infection (Fig. 4).

A majority of participants demonstrated awareness of the correlation between gestational age at the time of infection and the severity of fetal infection (88.4%; 273/309), as well as the possibility of congenital infection in the event of reinfection or reactivation of the virus (72.2%; 223/309). On the other hand, 67% of participants (207/309) were unaware of the existence of a treatment that could be administered to decrease the risk of maternal–fetal transmission or to improve neonatal prognosis in cases of fetal infection.

**Fig. 5:**
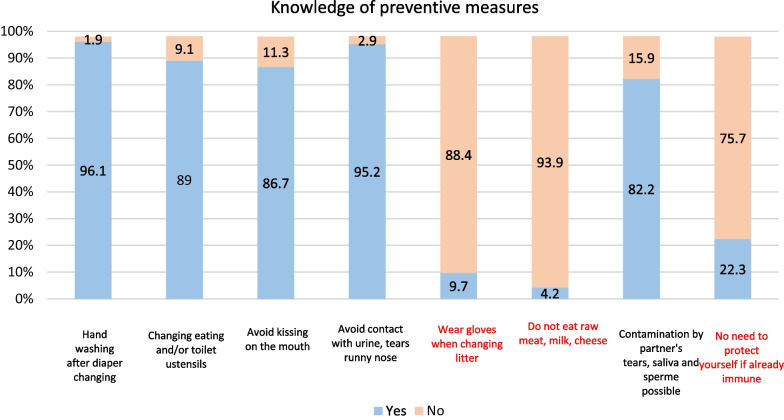
Knowledge on preventive measures. *Note* Incorrect answers are shown in red

Overall, 82% of participants were able to identify preventive hygiene measures to avoid CMV contamination. Washing hands after changing a baby (96.1%; 297/303) and avoiding contact with secretions (95.2%: 294/303) emerged as the two measures best known by the panel. However, the fact that these hygiene measures are also useful in women with immunity to avoid reinfection during pregnancy was the least known prevention advice (75.7%; 230/330). More than 85% of participants also correctly identified ineffective protective measures to avoid CMV infection (Fig. 5). The overall score regarding general knowledge of CMV was 31.5/42 (SD 4.6).

### Professional practice attitude

Table 2 shows the results of professional practices in relation to prevention and screening information. The results show that of the 300 practitioners who responded, 63.3% provided information about CMV to their pregnant patients (190/300). The majority report giving this information during the first consultation with the patient, either preconceptionally and/or in the 1st trimester (88.9%; 169/190). Among practitioners who reported not giving information on CMV to their patients, 23.6% justified this absence of information by their lack of virus knowledge (26/110). Regarding screening, the majority of participants reported offering it systematically (45.7%; 137/300) or for target groups (37.3%; 112/300). Among those who systematically offered it, the first consultation with the patient (either preconception or 1st trimester) was the period where it was most frequently carried out (96.3%; 132/137). For practitioners offering screening to target groups, the contact of their patient with young children (50.0%; 56/112) appeared to be the main reason for screening. The score assessing professional practice on four points revealed a median score of 3/4 (IQR 1–4).

**Table 2:** Professional practice regarding prevention and screening

	Total number of participants: N = 300
	Yes
	*n* (%)
Prevention	
Systematic prevention	190/300 (63.3)
1st consultation (pre/peri conception/1st trimester)	169/190 (88.9)
Pre conceptional	88/190 (46.3)
1st trimester	150/190 (78.9)
2nd trimester	36/190 (18.9)
3rd trimester	30/190 (15.8)
Post partum	21/190 (11.1)
Reasons for not providing information	110/300 (36.7)
Lack of time	13/110 (11.8)
Lack of knowledge on the subject	26/110 (23.6)
Rare virus	6/110 (5.5)
Forgotten	27/110 (24.5)
Not recommended	12/110 (10.9)
Screening	
Systematic screening	137/300 (45.7)
1st consultation (pre/peri conception/1st trimester)	132/137 (96.3)
Pre conceptional	42/137 (30.7)
1st trimester	123/137 (89.8)
2nd trimester	17/137 (12.4)
3rd trimester	7/137 (5.1)
Post partum	1/137 (0.7)
Target group for screening	112/300 (37.3)
After potential contamination	40/112 (35.7)
At the patient's request	39/112 (34.8)
If ultrasound sign	38/112 (33.9)
Seronegative patient	12/112 (10.7)
Patient in contact with young children	56/112 (50.0)
No screening	51/300 (17.0)

The answers in italics are those that allowed points to be obtained for the professional practice score

### Knowledge of CMV recommendations

The results relating to participants' knowledge and adherence to existing recommendations on CMV are presented in Table 3. A total of 62.0% of participants replied that they were aware of the existence of Swiss recommendations on the subject (181/292). Of these, 148 (81.8%) declared they had read the latest SSGO recommendations on CMV during pregnancy. Among them, prevention-related recommendations were well known and correctly identified (all pregnant women should be informed about CMV: 93.2%; 138/148, information to be given as soon as possible: 84.5%; 125/148). Regarding screening recommendations, 70.3% (104/148) of participants correctly identified that all pregnant patients should be informed of the possibility of CMV serological screening, and 59.5% (88/148) knew that this screening should be carried out as soon as possible if women wished. Concerning the management of patients with suspected infection, while 89.2% (132/148) of practitioners responded that they should refer their patients to a specialist in maternal–fetal medicine, gaps emerged in knowledge concerning the possibility of proposing the administration of antenatal treatment to reduce the risk of vertical transmission (49.3%; 73/148) or improve fetal prognosis in cases of congenital infection (48.7%; 72/148). The global score for the knowledge of the recommendations revealed a median score of 5/7 (IQR 4–6).

**Table 3:** Participants' knowledge of CMV recommendations

Knowledge of CMV recommendations	Total number of participants: N = 292
	Yes
	*n* (%)
Knowledge of Swiss CMV recommendations issued by the SSGO	148 (50.7)
What does this expert opinion recommend regarding prevention?	
All pregnant women should be informed about risks of CMV infection and hygiene measures	138/148 (93.2)
Only pregnant women with risk factors should be informed	7/148 (4.7)
This awareness-raising should be carried out as early as possible in pregnancy, as well as in the preconception period	125/148 (84.5)
What does this expert opinion recommend regarding screening?	
Offer screening only to pregnant women at risk	28/148 (18.9)
Inform all pregnant women or women wishing to become pregnant of the possibility of serological screening for CMV	104/148 (70.3)
Carry out screening as early as possible in pregnancy and, if possible, preconceptionally	88/148 (59.5)
What does this expert opinion recommend regarding management of suspected infections?	
All these situations should be referred to a specialist in maternal–fetal medicine	132/148 (89.2)
These situations can be followed up exclusively in the doctor's office if an ultrasound scan is performed there	8/148 (5.4)
Early prenatal treatment may be discussed to reduce the risk of vertical transmission	73/148 (49.3)
Prenatal treatment may be discussed to improve the outcome of an infected fetus	72/148 (48.7)

The answers in italics are those that allowed points to be obtained for the score on the knowledge of Swiss recommendations

### Identification of sociodemographic and professional factors influencing general knowledge of CMV

In a multivariable analysis, the following variables were independently associated with a higher score of general knowledge about CMV: having a higher level of education than the diploma needed (aCoeff 1.1 [0.0–2.1]; *p* = 0.023), practicing ultrasound (aCoeff 2.8 [1.3 to 4.3]; *p* =  < 0.001), preconception consultations (aCoeff 2.0 [0.6–3.3]; *p* = 0.017), participating in multidisciplinary symposia (aCoeff 1.1 [-0.1 to 2.2]; *p* = 0.021) and having received recent training on CMV (aCoeff 1.5 [0.5–2.5]; *p* < 0.001) (Table 4).

**Table 4:** Univariate and multivariate analyses to identify factors independently influencing general knowledge of CMV

Sociodemographic and professional factors	Score of knowledge n = 303					
	*n*	Mean (SD)	Univariate analysis		Multivariate analysis	
			Coeff [95% CI]	*p*	*a*Coeff [95% CI]	*p*
Gynecologists-Obstetricians	94 vs 209	33.9 (3.9) vs 30.3 (4.4)	3.6 [2.6 to 4.6]	**< 0.001**	− 0.5 [− 2.1 to 1.1]	0.561
General practitioners	5 vs 298	30.4 (2.7) vs 31.4 (4.6)	− 1.0 [− 5.0 to 3.0]	0.628		
Midwives	204 vs 99	30.3 (4.4) vs 33.7 (3.9)	− 3.4 [− 4.5 to − 2.4]	**< 0.001**	*	
Professional age > 10 years	189 vs 114	31.3 (4.5) vs 31.5 (4.6)	− 0.1 [− 1.2 to 0.9]	0.800		
Higher education^a^	161 vs 142	32.8 (4.4) vs 29.7 (4.2)	3.1 [2.1 to 4.1]	**< 0.001**	1.2 [0.2 to 2.2]	**0.022**
Private practice	115 vs 188	31.6 (4.6) vs 31.3 (4.5)	0.3 [− 0.7 to 1.4]	0.561		
University Hospital Centre	114 vs 189	31.6 (4.9) vs 31.2 (4.3)	0.4 [− 0.7 to 1.4]	0.503		
Peripheral hospitals	83 vs 220	31.4 (3.7) vs 31.4 (4.8)	0.1 [− 1.1 to 1.2]	0.876		
Private hospital	11 vs 292	31.5 (4.9) vs 31.4 (4.5)	0.1 [− 2.7 to 2.8]	0.956		
Birth center	12 vs 291	29.8 (4.1) vs 31.4 (4.6)	− 1.6 [− 4.2 to 1.0]	0.229		
Pregnancy follow-up	185 vs 118	31.7 (4.3) vs 30.8 (4.8)	0.9 [− 0.1 to 2.0]	**0.079**	− 0.6 [− 1.7 to 0.5]	0.285
Emergency ObGyn consultations	156 vs 147	32.0 (3.9) vs 30.7 (5.0)	1.4 [0.3 to 2.4]	**0.009**	0.2 [− 0.8 to 1.3]	0.666
Pre-conception consultations	69 vs 234	34.1 (3.9) vs 30.6 (4.4)	3.6 [2.4 to 4.8]	**< 0.001**	1.6 [0.2 to 3.0]	**0.022**
Ultrasound	94 vs 209	34.4 (3.8) vs 30.0 (4.2)	4.4 [3.4 to 5.4]	**< 0.001**	2.8 [1.3 to 4.3]	** < 0.001**
Birth preparation	102 vs 201	30.2 (4.2) vs 32.0 (4.6)	− 1.8 [− 2.8 to − 0.7]	**0.001**	− 0.3 [− 1.3 to 0.8]	0.625
Post partum	203 vs 100	30.7 (4.3) vs 32.8 (4.7)	− 2.0 [− 3.1 to − 1.0]	**< 0.001**	**	**
Childbirth	168 vs 135	31.6 (4.0) vs 31.1 (5.1)	0.5 [− 0.5 to 1.6]	0.307		
Attend multidisciplinary fetal medicine conferences	101 vs 202	33.4 (4.4) vs 30.3 (4.3)	3.1 [2.1 to 4.1]	**< 0.001**	1.2 [0.2 to 2.3]	**0.025**
Recent training	80 vs 223	33.1 (4.9) vs 30.7 (4.2)	2.3 [1.2 to 3.5]	**< 0.001**	1.7 [0.6 to 2.7]	**0.001**

^a^Than bachelor's degree and federal diploma in human medicine

*Not included in the multivariable analysis due to collinearity with GynObs

**Not included in the multivariable analysis due to collinearity with birth preparation

## Identification of sociodemographic and professional factors influencing the practice attitude

In a multivariable model, variables independently associated with a better score for professional practices were being a gynecologist-obstetrician (aCoeff 0.6 [0.0–1.1]; *p* = 0.037), having a high level of education (aCoeff 0.4 [0.0–0.7]; *p* = 0.038), practising pregnancy follow-up (aCoeff 0.9 [0.5–1.3]; *p* < 0.001) and attending symposia (aCoeff 0.6 [0.2–1]; *p* = 0.002) (Table 5).

**Table 5:** Univariate and multivariate analyses to identify factors independently influencing professional practices for CMV prevention

Sociodemographic and professional factors	Score of practice attitude n = 300						
	*n*	Median (IQR)	Univariate analysis			Multivariate analysis	
			Rank sum	*z*	*p*	*a*Coeff [95% CI]	*p*
Gynecologists-Obstetricians	93 vs 207	4 (3–4) vs 2 (0–4)	19,064.5	**− **7.6	**< 0.001**	0.6 [0.0 to 1.1]	**0.037**
General practitioners	5 vs 295	0 (0–1) vs 3 (1–4)					
Midwives	202 vs 98	2 (0–4) vs 4 (3–4)	25,822.5	6.8	**< 0.001**	*	
Professional age > 10 years	188 vs 112	3 (2–4) vs 2 (0–4)	30,121.5	**− **2.6	**0.008**	0.2 [**− **0.1 to 0.6]	0.192
Higher education^a^	160 vs 140	4 (2–4) vs 2 (0–4)	27,719.5	**− **5.1	**< 0.001**	0.4 [0.0 to 0.7]	**0.038**
Private practice	114 vs 186	3 (1–4) vs 2 (1–4)	18,442.5	**− **1.8	**0.060**	**− **0.3 [**− **0.7 to 0.0]	0.080
University Hospital Centre	113 vs 187	3 (0–4) vs 3 (1–4)	16,801	0.3	0.77		
Peripheral hospitals	82 vs 218	3 (2–4) vs 3 (1–4)	12,516.5	**− **0.3	0.78		
Private hospital	11 vs 289	4 (2–4) vs 3 (1–4)	1853	**− **0.7	0.46		
Birth center	12 vs 288	2 (2–4) vs 3 (1–4)	1814	**− **0.0	0.98		
Pregnancy follow-up	183 vs 117	4 (2–4) vs 1 (0–3)	32,509	**− **7.1	**< 0.001**	0.9 [0.5 to 1.3]	**< 0.001**
Emergency ObGyn consultations	155 vs 145	3 (2–4) vs 2 (0–4)	26,184.5	**− **4	**< 0.001**	0.0 [**− **0.4 to 0.4]	0.840
Preconception consultations	68 vs 232	4 (3–4) vs 2 (0–4)	14,100.5	**− **6.4	**< 0.001**	0.2 [**− **0.3 to 0.7]	0.395
Ultrasound	93 vs 207	4 (3–4) vs 2 (0–4)	18,628	**− **7	**< 0.001**	0.1 [**− **0.4 to 0.7]	0.571
Birth preparation	101 vs 199	2 (1–4) vs 3 (1–4)	14,192	1.5	0.14		
Post partum	201 vs 99	2 (1–4) vs 3 (1–4)	29,123.5	1.7	0.09	**	**
Childbirth	167 vs 133	3 (1–4) vs 2 (0–4)	26,487	**− **1.9	**0.06**	**− **0.0 [**− **0.4 to 0.4]	0.862
Attend multidisciplinary fetal medicine conferences	99 vs 201	4 (3–4) vs 2 (0–4)	19,362.5	**− **6.6	**< 0.001**	0.6 [0.2 to 1]	**0.002**
Recent training	80 vs 220	3 (1–4) vs 3 (1–4)	12,530	**− **0.8	0.44		

^a^Than bachelor's degree and federal diploma in human medicine

*Not included in the multivariable analysis due to collinearity with GynObs

**Not included in the multivariable analysis due to collinearity with Postpartum data

### Identification of sociodemographic and professional factors influencing the knowledge of national recommendations

In a multivariable model, it appeared that having a high level of education (aCoeff 0.7 [0.2–1.3]; *p* = 0.013), working in a university hospital center (aCoeff 0.8 [0.1– 1.4]; *p* = 0.015), and attending multidisciplinary fetal medicine symposia (aCoeff 0.5 [0.0–1.1]; *p* = 0.049) were independently associated with a better score of knowledge of the Swiss recommendations (Table 6).

**Table 6:** Univariate and multivariate analyses to identify factors independently influencing the knowledge of Swiss recommendations on CMV

Sociodemographic and professional factors	Score of recommendations knowledge n = 148						
	*n*	Median (IQR)	Univariate analysis			Multivariate analysis	
			Rank sum	*z*	*p*	*a*Coeff [95% CI]	*p*
Gynecologists-Obstetricians	75 vs 73	5 (4–6) vs 5 (3–6)	6193	− 2.4	**0.02**	− 0.0 [− 0.9 to 0.8]	0.922
General practitioners	1 vs 147	1 (1–1) vs 5 (4–6)					
Midwives	72 vs 76	5 (3–6) vs 5 (4–6)	4828.5	2.1	**0.04**	*	
Professional age > 10 years	99 vs 49	5 (4–6) vs 5 (3–6)	7152	0.9	0.35		
Higher education^a^	98 vs 50	5 (2–4) vs 2 (0–4)	8075	− 3.2	**0.001**	0.7 [0.2 to 1.3]	**0.013**
Private practice	70 vs 78	5 (4–6) vs 5 (4–7)	4996.5	0.9	0.39		
University Hospital Centre	51 vs 97	6 (4–7) vs 5 (4–6)	4359	− 2.3	**0.02**	0.8 [0.1 to 1.4]	**0.015**
Peripheral hospitals	37 vs 111	5 (4–6) vs 5 (4–6)	2551.5	0.9	0.36		
Private hospital	8 vs 140	5 (3–6) vs 5 (4–6)	521.5	0.6	0.53		
Birth center	4 vs 144	4.5 (3–5.5) vs 5 (4–6)	221.5	0.9	0.38		
Pregnancy follow-up	105 vs 43	5 (4–6) vs 5 (3–6)	8046	− 1	0.34		
Emergency ObGyn consultations	85 vs 63	5 (4–6) vs 5 (3–7)	6512	− 0.7	0.48		
Pre-conception consultations	54 vs 94	6 (5–6) vs 5 (3–6)	4627.5	− 2.5	**0.01**	0.5 [− 0.2 to 1.1]	0.141
Ultrasound	70 vs 78	5.5 (5–6) vs 5 (3–6)	5931	− 2.8	**0.00**	0.2 [− 0.5 to 1.0]	0.537
Birth preparation	44 vs 104	5 (3–6) vs 5 (4–6)	2698.5	2.5	**0.01**	− 0.3 [− 1 to 0.3]	0.334
Post partum	90 vs 58	5 (4–6) vs 5 (4–7)	6284.5	1.7	0.09		
Childbirth	87 vs 61	5 (4–6) vs 5 (4–6)	6428	0.2	0.83		
Attend multidisciplinary fetal medicine conferences	70 vs 78	6 (5–6) vs 5 (3–6)	5957.5	− 2.9	**0.00**	0.5 [0.0 to 1.1]	**0.049**
Recent training	51 vs 97	5 (4–6) vs 5 (4–6)	3727.5	0.3	0.77		

^a^Than bachelor's degree and federal diploma in human medicine

*Not included in the multivariable analysis due to collinearity with GynObs

### Correlation between CMV knowledge, recommendations and practices

Univariate analyses of the different outcomes to determine whether there was a correlation between them revealed that good knowledge of national recommendations had a significant positive influence on overall knowledge of CMV (coeff 0.2 [0.1—0.2]; *p* < 0.001), which in turn had a significant positive influence on good practice by healthcare professionals (coeff 0.7 [0.4–1.0]; *p* < 0.001).

## Discussion

### Interpretation

#### Knowledge of CMV

Our results showed that the knowledge of the perinatal healthcare professionals from French-speaking Switzerland surveyed highlighted significant gaps and was insufficient to enable optimal patient management. The literature has shown that a lack of CMV knowledge leads to insufficient patient awareness of the virus and a higher risk of seroconversion during pregnancy [10, 22, 23, 31, 39] but also to an increased risk of misdiagnosis, as well as a delay in the care of infected women, fetuses and newborns [22, 40]. The importance of women's knowledge of the virus and prevention was supported by the study by Vauloup-Fellous et al. [19], who determined that seroconversion could be reduced by a factor of 5 if hygiene measures were correctly applied. With a mean score of 31.5/42 (SD 4.6), the level of knowledge found in the present study is higher than the level highlighted by Pesch et al. in 2020 (17.5/36 (SD 6.4)) [41]. The good knowledge of maternal symptoms and protective hygiene measures as well as the low proportion of professionals able to identify the routes of CMV transmission were in line with previous studies [23, 30, 32, 41], although Swiss healthcare professionals appear to be more aware of the sexual transmission route [23, 30]. Nevertheless, 67% of participants were unaware of the existence of an off-label treatment to reduce the risk of maternal–fetal transmission or to improve neonatal prognosis in cases of fetal infection with mild to moderate inflammatory signs. Cordier et al. [23] noted that 20% of participants believed that effective treatment was available on the market, while Korver et al*.* [31] and Castillo et al. [42] highlighted that 34.6% and 14% of practitioners shared this belief, respectively.

Fellah et al*.* (2018) demonstrated an overall increase in knowledge of neonatal symptoms between 2012 and 2018 in France. While our results showed that hypotrophy was only known by 48.5% of participants, Fellah et al. (2018) reported a significant improvement in the knowledge of this symptom over 6 years in France (from 60 to 82.2%). While symptoms, such as microcephaly, are known at high rates between our study and that of Fellah et al. [23, 30] (> 80%), certain possible symptoms, such as petechiae, seizures and jaundice, are still not well known throughout the literature (< 40%). Among the long-term consequences, the risks of blindness, seizures and motor retardation are the least known to practitioners in all studies (< 57%) [23, 30]. One in five of the Swiss professionals surveyed have associated heart problems with CMV infections, both immediately and in the long term in children. The same tendency to misinterpret cardiac impairment as associated with cCMV was also highlighted by Muldoon et al*.* [32]. This result raises the question of a possible collective belief among Swiss professionals that CMV infection can damage the heart or of a mixture of knowledge with other pathologies that cause heart damage in newborns (such as parvovirus B19 or rubella). This example clearly illustrates the need not only for training but also for updating Swiss healthcare professionals' CMV infection knowledge and its consequences. Fellah et al. [30] hypothesized that national commitment to raising awareness of CMV education, along with updated recommendations from the French National College of Gynecologists and Obstetricians (CNGOF), could account for this improvement. The notable similarity between our knowledge’s results and those found by Fellah et al. [43] would suggest that the warning issued in 2018 by the Federal Office of Public Health (OFSP) on the lack of CMV-related national data has been heard. The publication in 2020 in the Swiss medical journal on the lack of recommendations on serological and neonatal screening in Switzerland may also have raised awareness among healthcare professionals in recent years [44]. In addition, Switzerland announced, in May 2019, the will to improve by 2024 population protection by strengthening the international system for the detection, surveillance, prevention and control of transmissible diseases [45]. The steps taken by healthcare decision-makers over the past 5 years may explain the results comparable to those found by Fellah et al. [30] after the 2018 awareness. Finally, healthcare providers might also have been sensitized to infectious disease during pregnancy due to the recent Zika, SARS-CoV-2 and Monkeypox pandemics.

Multivariate analyses determined that, surprisingly, profession was not a factor determining a good level of CMV knowledge, contrary to the results found by Cordier et al. [23]. However, higher education, providing ultrasounds or preconception consultations, participating in multidisciplinary symposia and having received recent training on CMV were associated with better overall CMV knowledge. Similar literature showed a crucial lack of contextualization of the authors' findings on knowledge with possible occupational and sociodemographic factors. Several authors have separated groups of participants by occupation to assess and compare CMV knowledge by professional sector [23, 30, 42]. Apart from identifying the groups most in need of being targeted for awareness-raising and training programs, this division nevertheless fails to identify the factors specifically associated with good CMV knowledge. Korver et al*.* and Cordier et al*.* [23, 31] determined that the level of knowledge increased with advanced professional experience as well as working in a prenatal diagnostic service. Muldoon et al*.* [32] were unable to identify any significant predictors of CMV knowledge due to a high degree of collinearity between the variables used in the model. However, they were able to highlight that although occupation was not correlated with better overall CMV knowledge, working in the field of infectious diseases nevertheless resulted in a higher knowledge score [32]. The study conducted in 2022 by Smither-Sheedy et al*.* [34] on the impact of continuing education via e-learning on healthcare professionals' CMV knowledge highlighted the fact that over 80% of participants reported never having received training on CMV and that continuing education significantly increased both knowledge and confidence among healthcare professionals. Fellah et al. [30] also argued that the implementation of educational programs could reinforce the impact of preventive measures and improve general knowledge of CMV infection [30]. Accordingly, promoting preconception consultations by midwives, advocating the widespread availability of training courses, making them attractive and financially accessible to all and, in line with the trend observed since the COVID-19 epidemic, encouraging home-based e-learning wherever possible, could be an interesting way of improving Swiss healthcare professionals' knowledge of CMV.

#### Professional practice

##### Prevention and timing

Our results showed that two-thirds of the professionals surveyed provide CMV counseling to their patients before or in early pregnancy. This result was similar to that found by Fellah et al*.* in 2018 but higher than those found in the rest of the literature [30, 41, 42]. The main reasons given by professionals who reported not systematically counseling were forgetfulness, lack of knowledge, lack of time, although it is not recommended and that CMV is a rare virus. In our study, lack of knowledge concerned a quarter of participants who did not advise their patients about CMV, whereas it concerned half of those surveyed by Castillo et al*.* [42]. However, they found the same trend concerning the lack of time and the fact that healthcare professionals thought it was not recommended by the guidelines. The lack of recommendations is a reason cited even more frequently in other studies [23, 30]. Some authors determined that the higher the level of knowledge, the more the preventive message is disseminated to patients [23]. It has been shown that the rate of counseling can increase after a 20-min training session on CMV [41].

##### Screening, timing and target groups

Almost half of the practitioners carried out systematic serological screening for CMV, and most of them did so during the first antenatal visit. Regarding nonroutine serological screening, caregivers reported targeting patients in contact with young children, in cases of maternal or fetal signs suggestive of CMV infection or in cases of patient request. In the literature, opinions diverge widely regarding systematic serological screening. In 2013, a review by Walker et al*.* determined that the answer to this question was neither "yes" nor "no" but "not yet", noting that the criteria in favor of such a practice had not yet been met, despite the definite advantages, mainly for seronegative patients [46]. In the absence of a vaccine and effective treatment, this ambivalence is due to the difficulty of interpreting CMV-specific antibodies and consequently to the wide margin of error in interpreting IgG and IgM levels and avidity, which can lead, e.g. to unjustified terminations of pregnancies [19, 22]. Another argument advanced concerns recent data showing that secondary infection with another strain of the virus is possible and that up to 1% of children born following secondary CMV infection will present with cCMV [47–49]. Between 2020 and 2021, two studies were published on the subject, taking opposing positions [22, 50]. Scientists in favor of systematic serological screening argue that the current treatment could reduce the risk of cCMV by up to 70% and that screening is easily accessible in middle- and high-income countries [50, 51]. Opponents highlight the risk of poor outcomes due to the lack of consistency of results and support the application of hygienic measures to decrease the rate of seroconversion during pregnancy [19, 22]. On a more measured note, Gievers et al*.* [52] propose targeted serological screening for at-risk populations of women. This ambivalence found in the literature was also reflected in our results. While half of the respondents decided to carry out systematic screening, those who opted for targeted screening selected patient groups also identified as at risk in the literature. The timing of screening was also in line with expert recommendations for early screening [14, 50]. However, in the absence of clear guidelines on screening, professional practices diverge, leaving room for personal judgment or maternal request to decide whether to carry out screening [53]. Ongoing scientific advances toward a reliable and effective treatment could enable clear guidelines to be drawn up, thereby promoting harmonization of practices for optimal care of pregnant patients.

The identification of sociodemographic and professional factors that could have an impact on the practices of healthcare professionals showed that being a gynecologist-obstetrician, having a high level of education, practicing pregnancy follow-up and attending symposia were associated with a better score for professional practices. As practices are difficult to define and therefore difficult to identify, very few researchers have analyzed the factors that might correlate with good professional practice [23]. At present, in Switzerland, in the absence of clear guidelines, CMV prevention and screening practices during pregnancy are mainly based on the experience, knowledge, beliefs and convictions of the caregiver in charge of the patient [53]. Our results assume that obstetricians and gynecologists who have their own patients and attend training courses and symposia regularly are those most likely to have good practice. However, this does not minimize the subjective aspect of what care patients do or do not receive, opening the door to inconsistent practices that vary by therapist, city or region and thus paving the way for inequality of care and treatment [54, 55]. The 2016 study on the heterogeneity of screening in Switzerland showed that 81% of patients in Geneva will be screened for CMV, compared with 17% in Zurich [54]. These results further underline the need to harmonize practices via clear guidelines and recommendations at the national level to offer women a consistent range and quality of care throughout the country.

#### Knowledge of recommendations

In our study, two-thirds of the participants were aware of the existence of national recommendations. As this proportion and the proportion of professionals who systematically provide preventive information are identical, this suggests that professionals who are aware of these recommendations and those who systematically deliver a preventive message are the same. These results contrast with those found in the literature, where the proportion of professionals aware of existing recommendations differs from one country to another. Indeed, Cordier et al*.* [23] showed that while 82% of professionals surveyed were aware of the existence of such recommendations in France, only 31% informed their patients of the measures to adopt to protect themselves. This rate had risen to 64% in 2018 [30]. Our results showed that prevention-related recommendations were much better known than those relating to screening and infection management. Put into perspective with current knowledge and literature, this can be explained by the fact that primary prevention through the application of protective hygiene measures is universally recommended [10, 18, 19, 22]. The ambivalence found in the literature concerning systematic serological screening and the type of management offered to infected patients may explain the lack of knowledge and divergence in professional practice concerning these aspects of the guidelines. The lack of knowledge and adherence to certain recommendations can also be explained by the level of evidence on which they are based. In Switzerland, the levels of evidence of CMV recommendations vary between Ib Ila and IIb and do not include a recommendation grade (A–B–C) [14]. These scientifically based Good Practice Recommendations (GPR) may be considered too weak by some professionals, which could explain the lack of interest in national CMV recommendations and their consequent heterogeneous implementation in practice. However, the results of this study showed that a high level of education, working in an academic environment and attending symposia were correlated with better knowledge of the recommendations. Regularly updated protocols in academic institutions probably had an impact on the knowledge score of professionals working in these institutions, as did the regular updating of knowledge through participation in symposia. In addition, our results showed that a good knowledge of the recommendations had a significant positive influence on CMV global knowledge, which in turn had a significant positive influence on professional practices. It is therefore essential to promote the existence of national guidelines to the entire target population of Swiss professionals and to increase adherence to them by continuing research into CMV prevention, screening and management to help improve the level of evidence on which the future guidelines will be based.

### Strengths and limitations

The subject of this article is a topical one, addressing a public health issue of major importance not only in Switzerland but also worldwide. This study is the first in Switzerland to examine the knowledge and attitudes of healthcare professionals about CMV infections during pregnancy. As such, it is a pioneering study that does not allow for an analysis of the evolution of healthcare professionals’ knowledge in recent years. However, as the study design was close to those already used in other countries, it was interesting to compare the level of knowledge as well as practice habits and adherence to recommendations with the results of other researchers. This enabled a more comprehensive analysis and understanding of French-speaking Swiss professionals' level of knowledge, as well as interactions with possible sociodemographic factors. Furthermore, given that only one-sixth of the eligible population participated in this study, there is an increased risk of non-response bias, which implies that the findings may not be representative because participants disproportionately possess certain characteristics that affect the results. In addition, it is possible that participants who felt they did not have sufficient knowledge about CMV did not take part in the study. This would lead to an overestimation of the knowledge and good practical attitudes found in our study. This hypothesis could also be reinforced by the fact that a certain number of participants responded to this study within two weeks of receiving CMV training and that part of the recruitment was carried out at specialized congresses. This may have induced a bias in the association between professional knowledge, level of training and participation in symposia. Regional limitations also need to be considered, as this study only covered the French-speaking region of Switzerland. As the divergence of national screening practices has been proven [54], it is possible that this aspect may have biased the results concerning the CMV screening practices of the professionals questioned. A comprehensive national study could provide a different perspective on the attitudes of healthcare professionals, enabling us to target prevention messages and recommendations to different regions and their needs.

### Recommendations

The results of this study highlight the current gaps in knowledge and practice regarding CMV in pregnancy among French-speaking Swiss professionals. There is an urgent need to raise practitioners' awareness of the importance of CMV knowledge to reduce the prevalence of CMV primary infections during pregnancy. Researchers who have investigated training impact on the knowledge and practice of professionals have determined that the latter significantly improves knowledge and, consequently, the practice and care of women [17, 24, 41, 56]. It would therefore be important to set up training programs for professionals working with pregnant women, regardless of their place of practice. Such training should be promoted not only at practice sites but also at congresses, training courses and professional groups/federations. As preconception consultations are an important factor correlated with good knowledge of CMV, consideration should be given to their inclusion and reimbursement, whether they are carried out by doctors or midwives in contact with patients during the pre- or periconceptional period. Moreover, our study showed that a good knowledge of the recommendations was significantly linked to a better overall CMV knowledge, thus having a significant impact on the good practice of healthcare providers. It is therefore necessary to pay particular attention not only to updating but also to promoting national recommendations relating to the virus and the management of pregnant patients. Indeed, as the level of evidence on which current recommendations are based is intermediate, it is vital to continue research into CMV, its management and future treatments to improve the level of evidence through well-conducted studies and thus be able to draw up GPR based on solid scientific data. The identification of factors with a positive influence on the variables of interest should also be considered by institutions to target professionals who are more likely to present deficits in knowledge and redirect them toward specific CMV training courses. The results of this study should also be considered by educational authorities, who should include more comprehensive CMV awareness and education in student curricula, to bring them into line with current recommendations as early as possible. Indeed, several studies have shown that students showed significant gaps in their knowledge of the virus at a very early stage in their studies [57]. Therefore, developing a specific course on CMV and making future healthcare professionals aware of the social and health aspects of cCMV infections is a national responsibility in the project to raise awareness of CMV infections during pregnancy.

## Conclusion

Our study confirms the significant gaps in overall knowledge of CMV among French-speaking Swiss healthcare professionals, as well as the heterogeneity of practices and lack of awareness of national recommendations on the subject. However, the identification of factors playing a role in improving knowledge and practices provides a solid basis for future research, which will not only improve knowledge by targeting certain professional profiles but also harmonize practices across the country and support the development of solid guidelines, thereby improving patient care.

### Supplementary Information

**Additional file 1.** Supplementary materiel for reviews.

## Data Availability

The dataset used and analyzed in this study is publicly available on the Synapse directory: 10.7303/syn52798260.1.

## Abbreviations

CMV: Cytomegalovirus
cCMV: Congenital cytomegalovirus
SNHL: Sensorineural hearing loss
SSGO: Swiss society of gynecology and obstetrics
HESAV: School of health sciences
GRSSGO: Group Romand of the Swiss society of gynecology and obstetrics
OFSP: Federal office of public health
GPR: Good practice recommendations
