# Supplementary material for: Cytomegalovirus infection during pregnancy: cross-sectional survey of knowledge and prevention practices of healthcare professionals in French-speaking Switzerland
Source: Virol J. 2024 Feb 21;21:45. doi: 10.1186/s12985-024-02318-w (PMC10882847; doi:10.1186/s12985-024-02318-w)
Supplement: Supplementary file 1 — Additional file 1. Supplementary materiel for reviews. [file 12985_2024_2318_MOESM1_ESM.pdf]

Supplementary materiel for reviews

Supp Table 1 Knowledge on transmission routes, potential symptoms and preventive measure to adopt

	Total number of participants			
	Yes		No	
	n	(%)	*n*	(%)
Main outcome: Professional knowledge				
Transmission route (***n* = 309) Multiple choice**				
Air	42/309	(13.6)	267/309	(86,4)
Sexual intercourse	130/309	(42.1)	179/309	(57.9)
Skin contact	84/309	(27.2)	225/309	(72.8)
Kiss	259/309	(83.8)	50/309	(16.2)
Blood	130/309	(42.1)	179/309	(57.9)
Baby’s diaper change	259/309	(83.8)	50/309	(16.2)
Breast feeding	90/309	(29.1)	219/309	(70.9)
I do not know	2/309	(0.7)	307/309	(99.4)
Maternal symptoms (***n* = 309) Multiple choice**				
Asymptomatic	267/309	(86.4)	42/309	(13.6)
Fever	200/309	(64.7)	109/309	(35.3)
Heart problems	4/309	(1.3)	305/309	(98.7)
Flu-like syndrome	258/309	(83.5)	51/309	(16.5)
Thrombosis	3/309	(1)	306/309	(99.0)
Deafness	6/309	(1.9)	303/309	(98.1)
Blindness	4/309	(1.3)	305/309	(98.7)
I do not know	4/309	(1.3)	305/309	(98.7)
Potential neonates clinical symptoms (***n* = 309) Multiple choice**				
Asymptomatic	193/309	(62.5)	116/309	(37.5)
Petechiae	87/309	(28.2)	222/309	(71.8)
Congenital cardiomyopathy	67/309	(21,7)	242/309	(78.3)
Microcephaly	264/309	(85.4)	45/309	(14.6)
Hypotrophy	150/309	(48.5)	159/309	(51.5)
Nephropathy	44/309	(14.2)	265/309	(85.8)
Macrosomia	2/309	(0.7)	307/309	(99.3)
Seizures	98/309	(31.7)	211/309	(68.3)
Anal atresia	3/309	(1)	306/309	(99.0)
Hearing loss	235/309	(76.1)	74/309	(23.9)
Icterus	83/309	(26.9)	226/309	(73.1)
I do not know	9/309	(2.9)	300/309	(97.1)
Possible long-term sequelae in newborns(***n* = 309) Multiple choice**				
Hearing loss	264/309	(85.4)	45/390	(14.6)
Mental retardation	249/309	(80.6)	60/309	(19.4)
Heart problems	53/309	(17.2)	256/309	(82.8)
Visual alteration	173/309	(56)	136/309	(44)
Seizures	90/309	(29.1)	219/309	(70.9)
Obesity	3/309	(1)	306/309	(99.0)
Motor delay	148/309	(47.9)	161/309	(52.1)
I do not know	13/309	(4.2)	296/309	(95.8)
Correlation between timing of infection ang gestational age (***n* = 309)**	273 /309	(88.3)	36/309	(11.7)
Can reinfection/reactivation imply congenital infection (***n* = 309)**	223/309	(72.2)	86/309	(27.8)
Is there an effective in utero treatment for infected fetuses? (***n* = 309)**	102/309	(33)	207/309	(67.0)
Knowledge of preventive measures (***n* = 303)**				
Hand washing after diaper change	297/303	(96.1)	6/303	(1.9)
Changing eating and/or toilet utensils	275/303	(89)	28/303	(9.1)
Avoid kissing on the mouth	268/303	(86.7)	35/303	(11.3)
Avoid contact with urine, tears, runny nose	294/303	(95.2)	9/303	(2.9)
Wear gloves when changing litter	30/303	(9.7)	273/303	(88.4)
Do not eat raw meat or raw milk cheese	13/303	(4.2)	290/303	(93.9)
Contamination by partner's tears, saliva and sperm possible	254/303	(82.2)	49/303	(15.9)
No need to protect yourself if already immune	69/303	(22.3)	234/303	(75.7)

Supplement material regarding participants CMV knowledge

Supp Figure 1a Percentage of correct answers for the virus transmission route

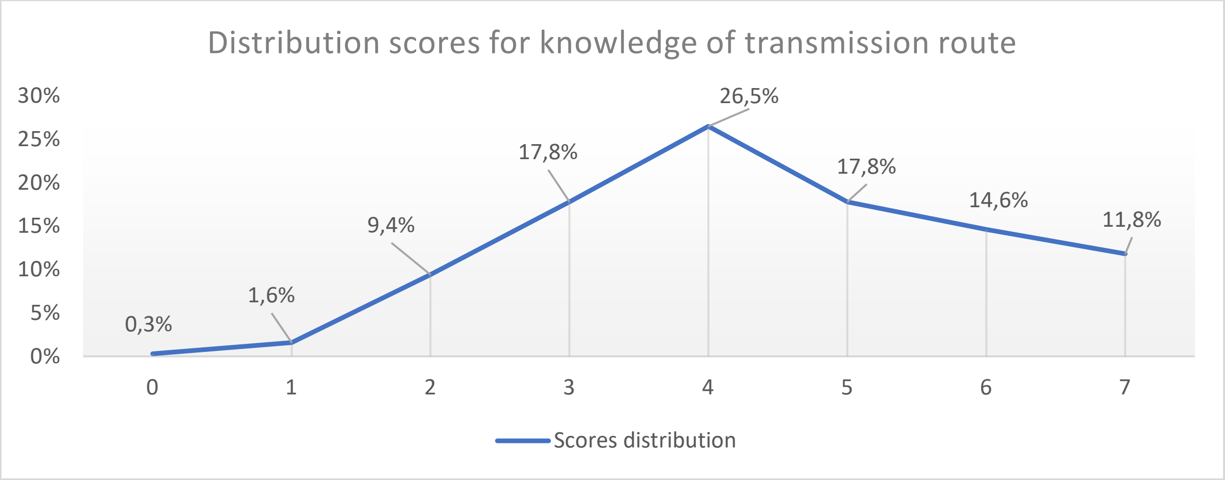

Supp Figure 1b Percentage of correct answers for the maternal clinical manifestations of CMV infection

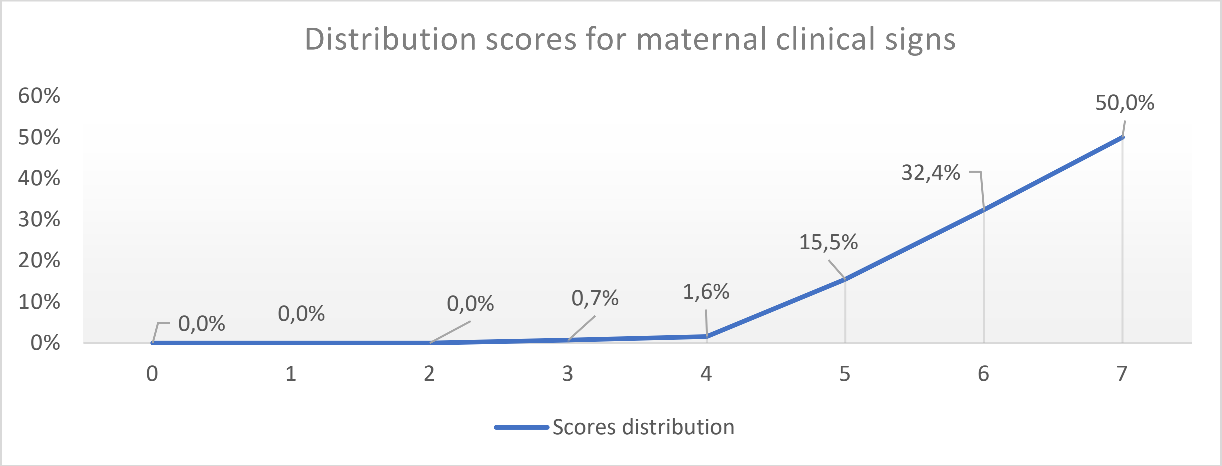

Supp Figure 1c Percentage of correct answers for the clinical signs in CMV-infected newborns

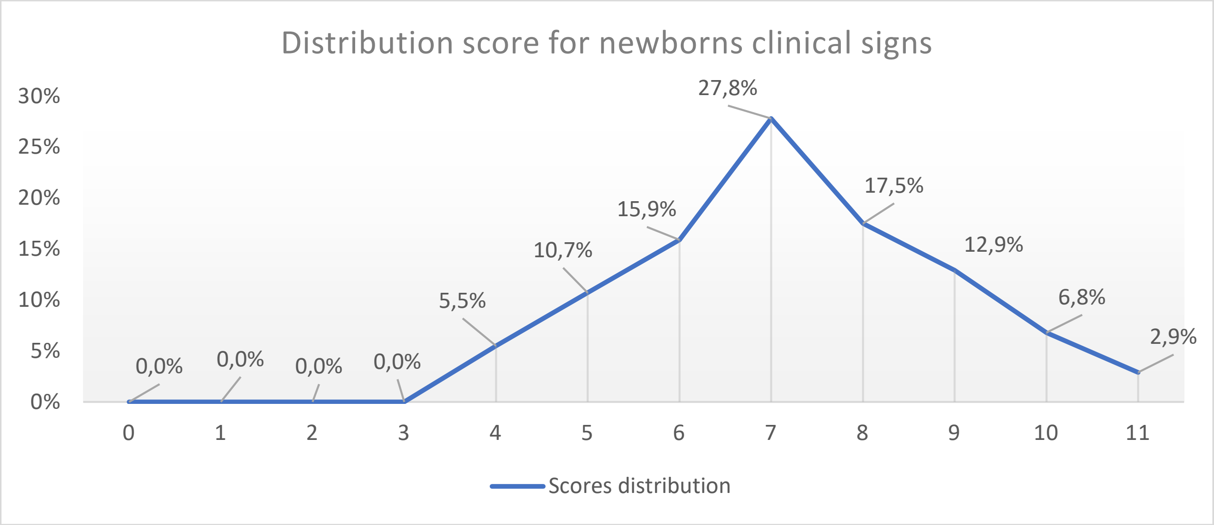

Supp Figure 1d Percentage of correct answers for the long-term sequelae of congenital CMV infections

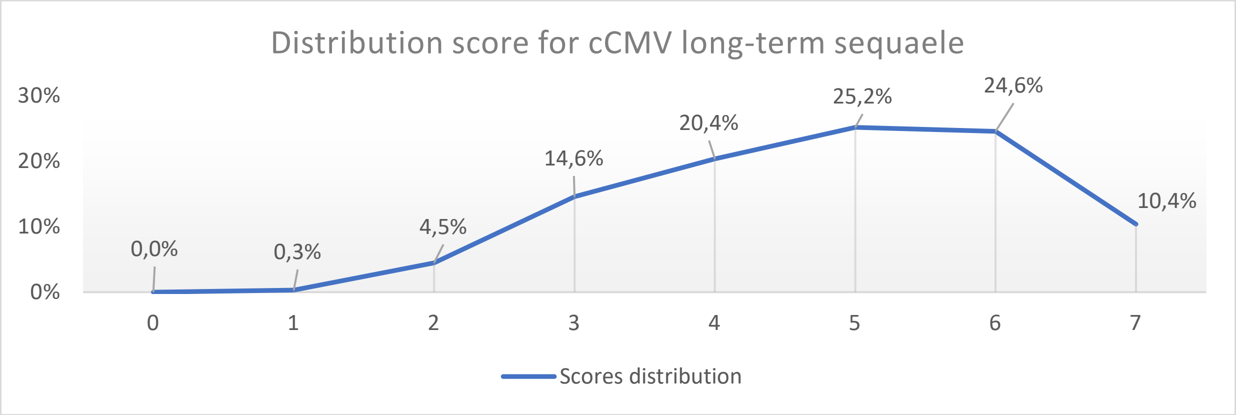

Supp Figure 1e Percentage of correct answers for the preventive hygiene measures

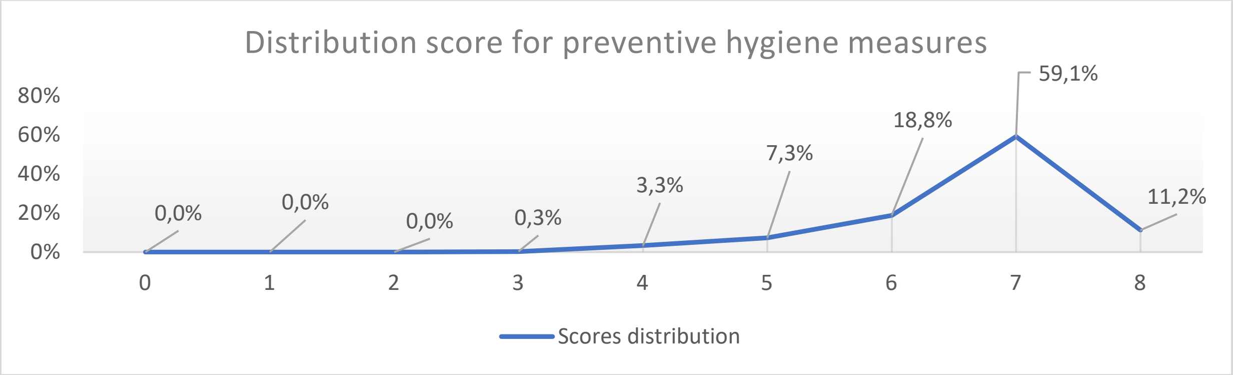

Supp Figure 1f Percentage of correct answers for healthcare professionals' knowledge of CMV

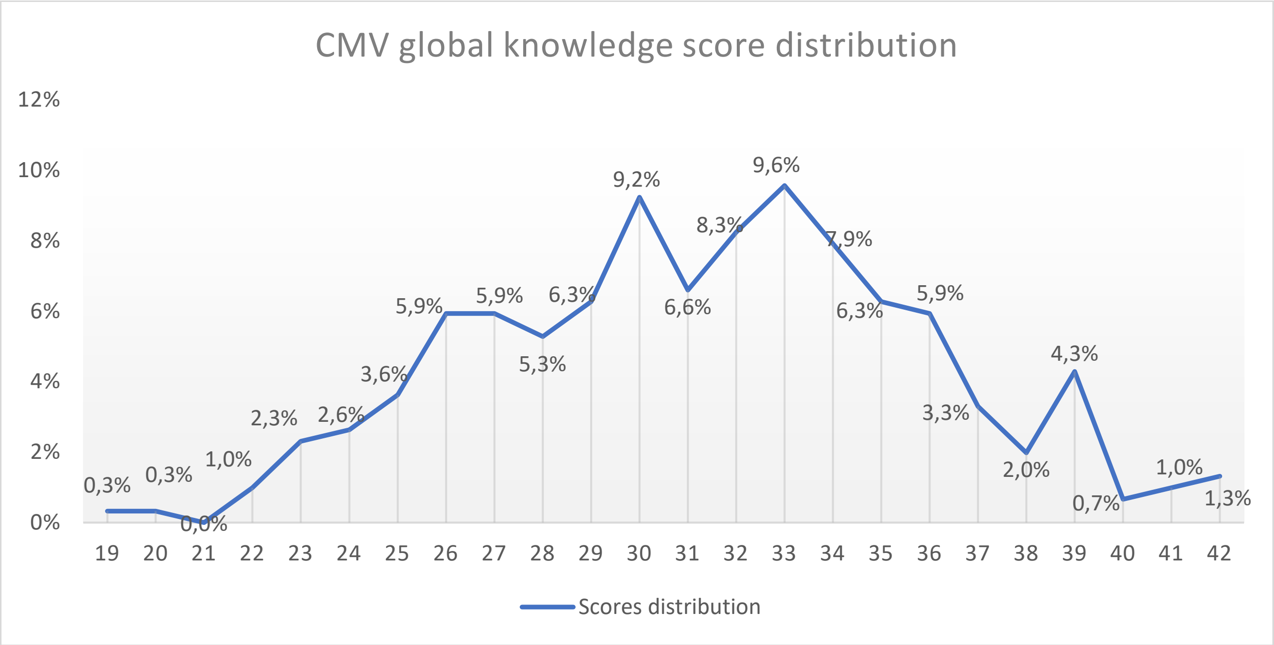

Supplement material regarding participants practice attitude

Supp Figure 2 Percentage of correct answers given by participants regarding the score for practice attitudes

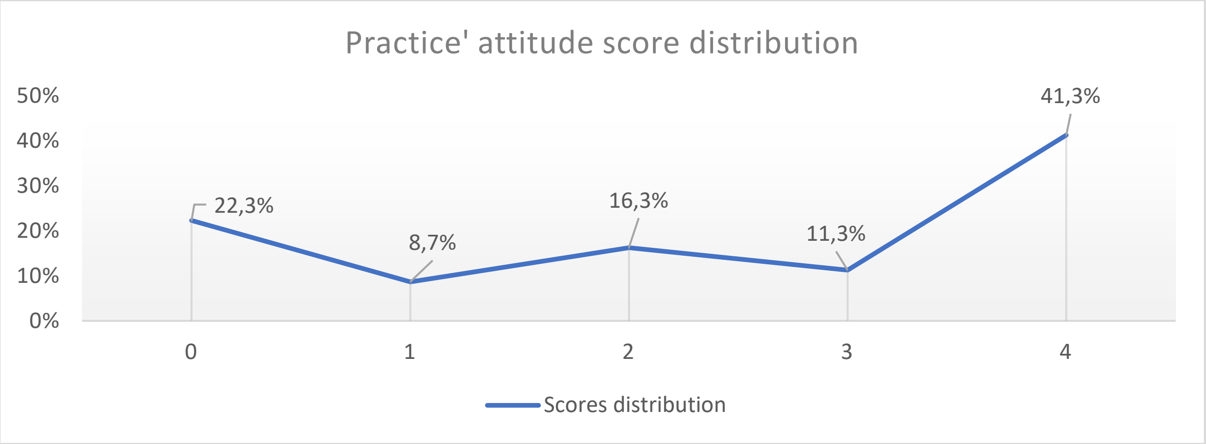

Supplement material regarding participants Swiss recommendations on CMV

Supp Figure 3 Percentage of correct answers regarding recommendations for the knowledge of the Swiss recommendations

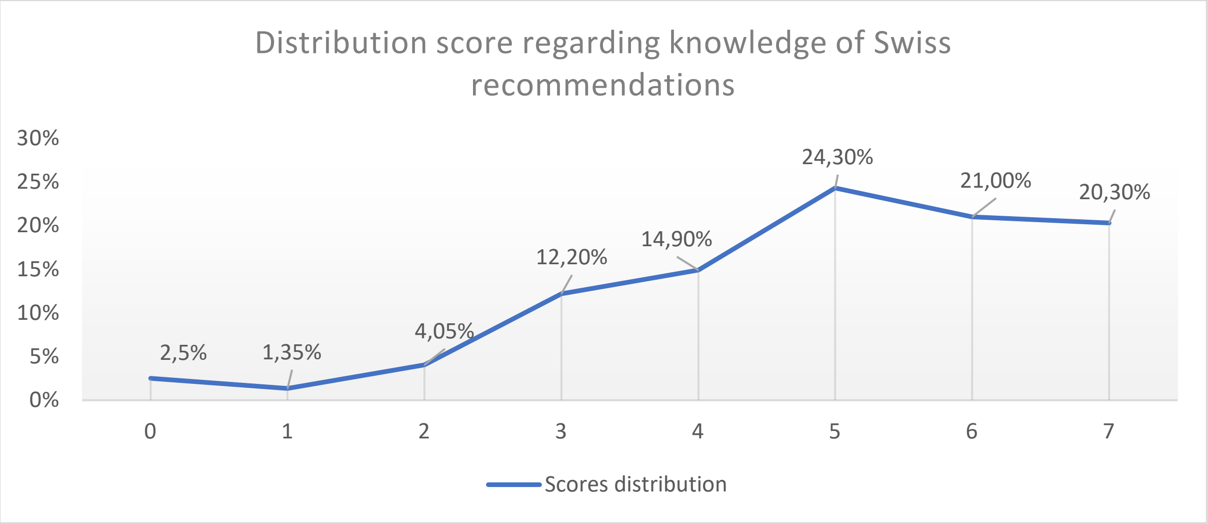

Supplement material: Questionnaire used for data collection

Connaissances et pratiques de prévention des professionnels de santé sur l'infection à Cytomégalovirus pendant la grossesse

Bonjour,

Dans le cadre d'une étude visant à évaluer les connaissances et les pratiques de prévention des professionnels de santé sur l'infection à Cytomégalovirus (CMV) pendant la grossesse, nous vous sollicitons pour remplir ce questionnaire. Sa durée est estimée à 5-10 minutes.

Cette étude se déroule en Suisse romande entre Octobre 2022 et Mai 2023, et vise à recruter 300 professionnels de la périnatalité (médecins ou sages-femmes).

La participation à cette enquête est facultative et anonyme. Vous pouvez arrêter à tout moment de remplir le questionnaire sans que vos données ne soient enregistrées. Les données anonymes seront sauvegardées sur un serveur institutionnel de HESAV en Suisse, et seront utilisées à des fins de recherche uniquement.

Si ce questionnaire venait à susciter des questions relatives au Cytomégalovirus, vous pourrez vous référer au flyer d'information transmis à la fin du questionnaire.

Pour toute question ou remarque concernant cette étude vous pouvez vous adresser à :

- Pauline Sartori, collaboratrice scientifique: pauline.sartori@hesav.ch

- Léo Pomar, professeur associé: leo.pomar@hesav.ch

Il y a 41 questions dans ce questionnaire.

Consentement

Souhaitez-vous participer à cette enquête? *

Veuillez sélectionner une réponse ci-dessous
Veuillez sélectionner une seule des propositions suivantes :

- ○ Oui
- ○ Non

Caractéristiques générales

Quelle est votre profession? *

Répondre à cette question seulement si les conditions suivantes sont réunies :
La réponse était 'Oui' à la question ' [consent]' (Souhaitez-vous participer à cette enquête?)

Cochez tout ce qui s’applique
Veuillez choisir toutes les réponses qui conviennent :

- ☐ Gynécologue-Obstétricien
- ☐ Médecin généraliste
- ☐ Sage-femme
- ☐ Autre: ____________

Quel est votre plus haut niveau de formation? *

Répondre à cette question seulement si les conditions suivantes sont réunies :
La réponse était 'Oui' à la question ' [consent]' (Souhaitez-vous participer à cette enquête?)

Veuillez sélectionner une réponse ci-dessous
Veuillez sélectionner une seule des propositions suivantes :

- ◯ Bachelor
- ◯ CAS ou diplôme universitaire de spécialisation
- ◯ Master
- ◯ Diplôme Fédéral en Médecine humaine
- ◯ Titre de spécialiste
- ◯ Titre de spécialiste avec formation approfondie en médecine foeto-maternelle
- ◯ Doctorat (MD et / ou PhD)
- ◯ Autre ____________

Depuis combien de temps êtes-vous diplômé(e) de votre formation initiale? (Diplôme fédéral de médecin, Bachelor de sage-femme, ou équivalents) *

Répondre à cette question seulement si les conditions suivantes sont réunies :
((consent.NAOK == 'AO01'))

Veuillez sélectionner une réponse ci-dessous
Veuillez sélectionner une seule des propositions suivantes :

- ◯ < 1 an
- ◯ 1 - 5 ans
- ◯ 6 - 10 ans
- ◯ > 10 ans

Dans quelle structure travaillez-vous? Merci de préciser le nom de l'institution si vous exercez à l'hôpital ou en clinique *

Répondre à cette question seulement si les conditions suivantes sont réunies :
((consent.NAOK == 'AO01'))

Ajoutez un commentaire seulement si vous sélectionnez la réponse.
Veuillez choisir toutes les réponses qui conviennent et laissez un commentaire :

- [ ] Cabinet libéral
- [ ] Hôpital universitaire
- [ ] Hôpital non-universitaire
- [ ] Clinique
- [ ] Maison de naissance
- [ ] Autre:

Quels actes réalisez-vous dans votre pratique professionnelle? *

Répondre à cette question seulement si les conditions suivantes sont réunies :
((consent.NAOK == 'AO01'))

Veuillez choisir toutes les réponses qui conviennent :

- [ ] Consultations de suivi de grossesse
- [ ] Consultations gynécologique et / ou obstétricales en urgence
- [ ] Consultations pré-conceptionnelles
- [ ] Echographies foetales de routine (1er et 2e trimestres)
- [ ] Echographies orientées (présentation foetale, croissance, localisation placentaire,...)
- [ ] Echographies foetales d'expertise
- [ ] Préparation à la naissance
- [ ] Accouchements
- [ ] Suivi du post-partum
- [ ] Autre: ____________________

Ceci est une texte d’aide pour la question.

Assistez-vous à des colloques multidisciplinaires de médecine foetale? *

Répondre à cette question seulement si les conditions suivantes sont réunies :
La réponse était 'Oui' à la question ' [consent]' (Souhaitez-vous participer à cette enquête?)

Veuillez sélectionner une seule des propositions suivantes :

- ○ Oui
- ○ Non

Connaissances sur le CMV

Connaissances sur le CMV

D'après vous, comment le CMV peut-il se transmettre ? *

Répondre à cette question seulement si les conditions suivantes sont réunies :
La réponse était 'Oui' à la question ' [consent]' (Souhaitez-vous participer à cette enquête?)

Cochez tout ce qui s’applique
Veuillez choisir toutes les réponses qui conviennent :

- [ ] Par l'air
- [ ] Lors de rapports sexuels
- [ ] Par contact cutané
- [ ] Par un baiser
- [ ] Par le sang
- [ ] Lors du change du bébé
- [ ] L'allaitement
- [ ] Je ne sais pas

Quelles sont les manifestations cliniques maternelles, lors d'une infection à CMV ? *

Répondre à cette question seulement si les conditions suivantes sont réunies :
La réponse était 'Oui' à la question ' [consent]' (Souhaitez-vous participer à cette enquête?)

Cochez tout ce qui s’applique
Veuillez choisir toutes les réponses qui conviennent :

- [ ] Asymptomatique
- [ ] Fièvre
- [ ] Problème cardiaque
- [ ] Syndrome pseudo-grippal
- [ ] Thrombose
- [ ] Surdité
- [ ] Cécité
- [ ] Je ne sais pas

Quels sont les symptômes possibles chez un nouveau-né infecté par le CMV ? *

Répondre à cette question seulement si les conditions suivantes sont réunies :
La réponse était 'Oui' à la question ' [consent]' (Souhaitez-vous participer à cette enquête?)

Cochez tout ce qui s’applique
Veuillez choisir toutes les réponses qui conviennent :

- ☐ Asymptomatique
- ☐ Pétéchies
- ☐ Cardiomyopathie congénitale
- ☐ Microcéphalie
- ☐ Hypotrophie
- ☐ Néphropathie
- ☐ Macrosomie
- ☐ Convulsions
- ☐ Atrésie anale
- ☐ Perte auditive
- ☐ Ictère
- ☐ Je ne sais pas

Quels sont les effets possibles sur le long terme en cas d'infection congénitale au CMV? *

Répondre à cette question seulement si les conditions suivantes sont réunies :
La réponse était 'Oui' à la question ' [consent]' (Souhaitez-vous participer à cette enquête?)

Cochez tout ce qui s'applique
Veuillez choisir toutes les réponses qui conviennent :

- ☐ Perte de l'audition
- ☐ Retard mental
- ☐ Problème cardiaque
- ☐ Altération visuelle
- ☐ Autisme
- ☐ Convulsions
- ☐ Obésité
- ☐ Retard moteur
- ☐ Je ne sais pas

Selon vous, la sévérité de l'atteinte foetale varie-t-elle avec le terme de l'infection maternelle ? *

Répondre à cette question seulement si les conditions suivantes sont réunies :
La réponse était 'Oui' à la question ' [consent]' (Souhaitez-vous participer à cette enquête?)

Veuillez sélectionner une réponse ci-dessous
Veuillez sélectionner une seule des propositions suivantes :

- ○ Oui
- ○ Non
- ○ Je ne sais pas

A votre avis, une réinfection/réactivation peut-elle entraîner une infection congénitale ? *

Veuillez sélectionner une réponse ci-dessous
Veuillez sélectionner une seule des propositions suivantes :

- ◯ Oui
- ◯ Non
- ◯ Je ne sais pas

Selon vous, existe-t-il des traitements in utero pour le foetus infecté au CMV ayant prouvé leur efficacité ? *

Veuillez sélectionner une réponse ci-dessous
Veuillez sélectionner une seule des propositions suivantes :

- ◯ Oui
- ◯ Non
- ◯ Je ne sais pas

Prévention du CMV

Parmi la liste ci-dessous, quelles sont les propositions qui appartiennent à la prévention du CMV?

Pour chaque mesure choisie, veuillez indiquer si elle vous semble applicable: *Très facilement, plutôt facilement, plutôt difficilement, très difficilement.*

Le lavage soigneux des mains après le change d’un enfant permet-il de se protéger contre le CMV? *

Répondre à cette question seulement si les conditions suivantes sont réunies :
La réponse était 'Oui' à la question ' [consent]' (Souhaitez-vous participer à cette enquête?)

Veuillez sélectionner une seule des propositions suivantes :

- ◯ Oui
- ◯ Non

Cette mesure vous semble applicable: *

Répondre à cette question seulement si les conditions suivantes sont réunies :
La réponse était 'Oui' à la question ' [lavagemains]' (Le lavage soigneux des mains après le change d’un enfant permet-il de se protéger contre le CMV?)

Veuillez sélectionner une réponse ci-dessous
Veuillez sélectionner une seule des propositions suivantes :

- ◯ Très facilement
- ◯ Plutôt facilement
- ◯ Plutôt difficilement
- ◯ Très difficilement

Pour se protéger du CMV il ne faut pas utiliser soi-même les ustensiles de repas ou de toilette d’un enfant: *

Répondre à cette question seulement si les conditions suivantes sont réunies :
La réponse était 'Oui' à la question ' [consent]' (Souhaitez-vous participer à cette enquête?)

Veuillez sélectionner une seule des propositions suivantes :

- ◯ Oui
- ◯ Non

Cette mesure vous semble applicable: *

Répondre à cette question seulement si les conditions suivantes sont réunies :
La réponse était 'Oui' à la question ' [ustensile]' (Pour se protéger du CMV il ne faut pas utiliser soi-même les ustensiles de repas ou de toilette d’un enfant:)

Veuillez sélectionner une réponse ci-dessous
Veuillez sélectionner une seule des propositions suivantes :

- ◯ Très facilement
- ◯ Plutôt facilement
- ◯ Plutôt difficilement
- ◯ Très difficilement

Eviter d’embrasser un enfant sur la bouche, permet-il de se protéger contre le CMV? *

Répondre à cette question seulement si les conditions suivantes sont réunies :
La réponse était 'Oui' à la question ' [consent]' (Souhaitez-vous participer à cette enquête?)

Veuillez sélectionner une seule des propositions suivantes :

- ◯ Oui
- ◯ Non

Cette mesure vous semble applicable : *

Répondre à cette question seulement si les conditions suivantes sont réunies :
La réponse était 'Oui' à la question ' [bisou]' (Eviter d’embrasser un enfant sur la bouche, permet-il de se protéger contre le CMV?)

Veuillez sélectionner une réponse ci-dessous
Veuillez sélectionner une seule des propositions suivantes :

- ◯ Très facilement
- ◯ Plutôt facilement
- ◯ Plutôt difficilement
- ◯ Très difficilement

Eviter d'être en contact avec les urines, les larmes ou le nez qui coule d'un enfant permet de se protéger du CMV: *

Répondre à cette question seulement si les conditions suivantes sont réunies :
La réponse était 'Oui' à la question ' [consent]' (Souhaitez-vous participer à cette enquête?)

Veuillez sélectionner une seule des propositions suivantes :

- ◯ Oui
- ◯ Non

Cette mesure vous semble applicable : *

Répondre à cette question seulement si les conditions suivantes sont réunies :
La réponse était 'Oui' à la question ' [urine]' (Eviter d'être en contact avec les urines, les larmes ou le nez qui coule d'un enfant permet de se protéger du CMV:)

Veuillez sélectionner une réponse ci-dessous
Veuillez sélectionner une seule des propositions suivantes :

- ◯ Très facilement
- ◯ Plutôt facilement
- ◯ Plutôt difficilement
- ◯ Très difficilement

Utiliser des gants pour nettoyer la litière du chat permet de se protéger du CMV? *

Répondre à cette question seulement si les conditions suivantes sont réunies :
La réponse était 'Oui' à la question ' [consent]' (Souhaitez-vous participer à cette enquête?)

Veuillez sélectionner une seule des propositions suivantes :

◯ Oui
◯ Non

Cette mesure vous semble applicable : *

Répondre à cette question seulement si les conditions suivantes sont réunies :
((litiere.NAOK == 'Y'))

Veuillez sélectionner une réponse ci-dessous
Veuillez sélectionner une seule des propositions suivantes :

◯ Très facilement
◯ Plutôt facilement
◯ Plutôt difficilement
◯ Très difficilement

Ne pas manger de viande crue ou de fromage au lait cru permet de se protéger du CMV *

Répondre à cette question seulement si les conditions suivantes sont réunies :
La réponse était 'Oui' à la question ' [consent]' (Souhaitez-vous participer à cette enquête?)

Veuillez sélectionner une seule des propositions suivantes :

- ◯ Oui
- ◯ Non

Cette mesure vous semble applicable: *

Répondre à cette question seulement si les conditions suivantes sont réunies :
((viande.NAOK == 'Y'))

Veuillez sélectionner une réponse ci-dessous
Veuillez sélectionner une seule des propositions suivantes :

- ◯ Très facilement
- ◯ Plutôt facilement
- ◯ Plutôt difficilement
- ◯ Très difficilement

Le partenaire d'une femme enceinte peut également lui transmettre le CMV par les liquides biologiques (salive, larme, sperme) s'il est infecté: *

Répondre à cette question seulement si les conditions suivantes sont réunies :
La réponse était 'Oui' à la question ' [consent]' (Souhaitez-vous participer à cette enquête?)

Veuillez sélectionner une seule des propositions suivantes :

- ○ Oui
- ○ Non

Demander au partenaire de respecter des règles d'hygiène similaires vous semble applicable : *

Répondre à cette question seulement si les conditions suivantes sont réunies :
((partenaire.NAOK == 'Y'))

Veuillez sélectionner une réponse ci-dessous
Veuillez sélectionner une seule des propositions suivantes :

- ○ Très facilement
- ○ Plutôt facilement
- ○ Plutôt difficilement
- ○ Très difficilement

Il est inutile de se protéger du CMV pendant la grossesse si on est immunisé *

Répondre à cette question seulement si les conditions suivantes sont réunies :
La réponse était 'Oui' à la question ' [consent]' (Souhaitez-vous participer à cette enquête?)

Veuillez sélectionner une seule des propositions suivantes :

- ◯ Oui
- ◯ Non

A propos des pratiques

Donnez-vous des informations aux femmes enceintes sur le CMV ? *

Répondre à cette question seulement si les conditions suivantes sont réunies :
La réponse était 'Oui' à la question ' [consent]' (Souhaitez-vous participer à cette enquête?)

Veuillez sélectionner une seule des propositions suivantes :

- ◯ Oui
- ◯ Non

Si oui, à quel terme donnez-vous l'information ? *

Répondre à cette question seulement si les conditions suivantes sont réunies :
La réponse était 'Oui' à la question ' [info]' (Donnez-vous des informations aux femmes enceintes sur le CMV ?)

Cochez tout ce qui s’applique
Veuillez choisir toutes les réponses qui conviennent :

- [ ] pré-conceptionnel
- [ ] 1er trimestre
- [ ] 2ème trimestre
- [ ] 3ème trimestre
- [ ] post-partum
- [ ] Autre: ____________

Si non, pourquoi ? *

Répondre à cette question seulement si les conditions suivantes sont réunies :
La réponse était 'Non' à la question ' [info]' (Donnez-vous des informations aux femmes enceintes sur le CMV ?)

Cochez tout ce qui s’applique
Veuillez choisir toutes les réponses qui conviennent :

- [ ] Manque de temps
- [ ] Manque d'information
- [ ] C'est un virus rare
- [ ] J'oublie souvent
- [ ] Cela n'est pas recommandé
- [ ] Autre: ____________

Proposez-vous à chaque patiente enceinte le dépistage du CMV pendant la grossesse ? *

Répondre à cette question seulement si les conditions suivantes sont réunies :
La réponse était 'Oui' à la question ' [consent]' (Souhaitez-vous participer à cette enquête?)

Veuillez sélectionner une seule des propositions suivantes :

- ◯ Oui
- ◯ Non

Si oui, à quel moment le proposez-vous ? *

Répondre à cette question seulement si les conditions suivantes sont réunies :
La réponse était 'Oui' à la question ' [depistage]' (Proposez-vous à chaque patiente enceinte le dépistage du CMV pendant la grossesse ?)

Cochez tout ce qui s’applique
Veuillez choisir toutes les réponses qui conviennent :

- ☐ pré-conceptionnel
- ☐ 1er Trimestre
- ☐ 2ème Trimestre
- ☐ 3ème Trimestre
- ☐ Post-partum
- ☐ Autre: ____________

Si non, à qui le proposez-vous ? *

Répondre à cette question seulement si les conditions suivantes sont réunies :
La réponse était 'Non' à la question ' [depistage]' (Proposez-vous à chaque patiente enceinte le dépistage du CMV pendant la grossesse ?)

Cochez tout ce qui s'applique
Veuillez choisir toutes les réponses qui conviennent :

- ☐ Après un potentiel contage
- ☐ A la demande de la patiente
- ☐ Devant des signes échographiques
- ☐ Si la patiente est séronégative
- ☐ Aux patientes régulièrement en contact avec des enfants en bas âge (femme travaillant au contact d'enfants en bas âge, femme ayant un enfant en bas âge...)
- ☐ Je ne le fais pas
- ☐ Autre: ____________

Connaissances des recommandations

Savez-vous s'il existe des recommandations Suisses concernant le dépistage et la prévention du CMV chez la femme enceinte ? *

Répondre à cette question seulement si les conditions suivantes sont réunies :
La réponse était 'Oui' à la question ' [consent]' (Souhaitez-vous participer à cette enquête?)

Veuillez sélectionner une réponse ci-dessous
Veuillez sélectionner une seule des propositions suivantes :

- ◯ Oui
- ◯ Non
- ◯ Je ne sais pas

Avez-vous pris connaissance de l'avis d'expert de la Société Suisse de Gynécologie Obstétrique sur le Cytomégalovirus? (Avis d'expert n°73 de la SSGO) *

Répondre à cette question seulement si les conditions suivantes sont réunies :
((recocmv.NAOK == "AO01") or (recocmv.NAOK == "AO02") or (recocmv.NAOK == "AO03"))

Veuillez sélectionner une réponse ci-dessous
Veuillez sélectionner une seule des propositions suivantes :

- ◯ Oui
- ◯ Non
- ◯ Approximativement

Selon vous, quelles-sont les recommandations de cet avis d'expert en terme de prévention primaire?

Répondre à cette question seulement si les conditions suivantes sont réunies :
((recossgo.NAOK == "oui") or (recossgo.NAOK == "."))

Cochez tout ce qui s’applique
Veuillez choisir toutes les réponses qui conviennent :

- ☐ Toutes les femmes enceintes devraient être informées sur les risques d’infection à CMV et sur les mesures d’hygiène
- ☐ Uniquement les femmes enceintes avec facteur de risque (contact étroit avec des jeunes enfants < 4 ans) devraient être informées sur les risques d’infection à CMV et sur les mesures d’hygiène
- ☐ Cette sensibilisation devrait être réalisée le plus tôt possible pendant la grossesse, ainsi qu'en période pré-conceptionnelle

Selon vous, quelles-sont les recommandations de cet avis d'experts en terme de dépistage?

Répondre à cette question seulement si les conditions suivantes sont réunies :
((recossgo.NAOK == "oui") or (recossgo.NAOK == "."))

Cochez tout ce qui s’applique
Veuillez choisir toutes les réponses qui conviennent :

☐ Proposer un dépistage uniquement aux femmes enceintes à risque (contact étroit avec des jeunes enfants < 4 ans)

☐ Informer toutes les femmes enceintes ou souhaitant une grossesse de la possibilité de réaliser un dépistage sérologique du CMV

☐ Réaliser ce dépistage le plus tôt possible pendant la grossesse et si possible en pré-conceptionnel

Selon vous, quelles-sont les recommandations de cet avis d'experts en terme de prise en charge des suspicions d'infection à CMV pendant la grossesse?

Répondre à cette question seulement si les conditions suivantes sont réunies :
((recossgo.NAOK == "oui") or (recossgo.NAOK == "."))

Cochez tout ce qui s’applique
Veuillez choisir toutes les réponses qui conviennent :

☐ Toutes ces situations devrait être adressées à un spécialiste en médecine foeto-maternelle

☐ Ces situations peuvent être suivies exclusivement en cabinet de ville si une échographie y est réalisée

☐ Un traitement prénatal précoce pourra être discuté pour diminuer le risque de transmission verticale

☐ Un traitement prénatal pourra être discuté pour améliorer l’issue d’un foetus infecté

Commentaire

Avez-vous une remarque ou un commentaire relatif à cette étude ou à la prévention du Cytomégalovirus chez la femme enceinte?

Veuillez écrire votre réponse ici :

<u>Résumé de l'avis d'experts n°73 de la SSGO:</u>
L'infection congénitale à CMV est la cause la plus fréquente de malformations et de troubles du développement congénitaux d'origine infectieuse.
Un contact étroit avec des jeunes enfants < 4 ans constitue le principal facteur de risque de séroconversion maternelle.

Le risque de transmission verticale est relativement élevé et dépend de l'âge gestationnel. Selon les données actuelles, une transmission jusqu'à 12 à 14 SA expose le foetus à un risque élevé de séquelles, alors que ce risque semble moindre lors d'une transmission plus tardive.
Toutes les femmes enceintes ou envisageant une grossesse doivent recevoir dès que possible des informations sur les risques d'infection à CMV et des recommandations portant sur les mesures d'hygiène préventives visant à diminuer ce risque. Par ailleurs elles devraient être informées de la possibilité d'évaluer le statut sérologique au CMV avant la conception et au début de la grossesse. Les avantages et désavantages d'avoir connaissance du statut sérologique doivent être discutés pour permettre une décision éclairée. Ceci doit également inclure le risque individuel d'infection à CMV de la femme (contact avec des enfants < 4 ans à la maison ou au travail, ou exposition présumée).
En cas (de suspicion) d'infection à CMV pendant la grossesse, la prise en charge devrait être assurée par un spécialiste en médecine foeto-maternelle. Selon des études récentes, il existe des options thérapeutiques efficaces pour réduire le risque de transmission verticale au début de la grossesse ainsi que pour améliorer l'issue d'un foetus infecté.
Une interdiction de travail généralisée pour la femme enceinte à risque n'est pas recommandée et est uniquement indiquée si les mesures d'hygiène nécessaires ne peuvent pas être respectées pour des raisons opérationnelles. Ceci peut être limité au premier trimestre de la grossesse.
En dernier lieu il convient de procéder à une évaluation du nouveau-né en cas d'infection à CMV suspectée ou prouvée pendant la grossesse (confirmation de l'infection à CMV par PCR urinaire dans les 3 premières semaines de vie) par un néonatologue ou un pédiatre.

Vous pouvez retrouver cet avis d'expert
içi: 73_Cytomegalievirus_und_Schwangerschaft_F_aktualisiert.pdf (sggg.ch)

Merci pour votre aimable participation. Nous restons à votre disposition pour répondre à toute question.

Contact: leo.pomar@hesav.ch

16/01/2024 – 12:31
Envoyer votre questionnaire.
Merci d’avoir complété ce questionnaire.

<u>Supplement material: Expert opinion of the Swiss Society of Gynaecology and Obstetrics on the prevention of Cytomegalovirus</u>

Avis d'experts Nº 73 (remplace le N°47)

Commission Assurance Qualité

Président Prof. Dr Daniel Surbek

Cytomégalovirus (CMV) et grossesse

L. Schäffer, N. Ochsenbein, M. Boulvain, D. Baud, L. Raio, A. Duppenthaler[a], B. Martinez de Tejada, S. Iff[b], S. Tercanli, D. Surbek

Académie de médecine fœto-maternelle, SSGO
[a]Infectiologie pédiatrique, Inselspital Berne, [b]Secrétariat d'Etat à l'Economie SECO

	Niveau de preuve
**Introduction**<br>L'infection congénitale à CMV est la cause la plus fréquente de malformations et de troubles du développement congénitaux d'origine infectieuse (principalement surdité et retard psychomoteur) et peut entraîner le décès du fœtus ou du nouveau-né.<br>Dans le cas d'une primo-infection maternelle, les taux de transmission intra-utérins dépendent de l'âge gestationnel : 5 % environ préconceptionnel (jusqu'à trois mois), 21 % périconceptionnel (de 4 semaines avant à 6 semaines après les dernières règles), 37 % au premier trimestre et jusqu'à 66 % au troisième trimestre. Une réactivation et une réinfection à CMV (non primo-infection) peuvent également provoquer une infection intra-utérine. Les taux exacts de transmission verticale des infections secondaires ne sont pas connus, en raison de la difficulté à définir et diagnostiquer une infection secondaire. Toutefois le risque, estimé à 0,2-3,4 %, semble être significativement plus faible que pour la primo-infection. Les lésions congénitales sont les plus graves en cas d'infection pendant le premier trimestre et des études récentes suggèrent qu'il n'y a un risque d'atteinte neurologique qu'en cas d'infection au premier trimestre. De même, une perte auditive se produit principalement après une infection au premier trimestre. Des lésions ont été décrites, bien que rarement, après une infection tardive, et l'éventualité d'une possible erreur de classification est discutée. La transmission intra-utérine n'entraîne pas toujours des lésions congénitales, mais des anomalies neurologiques significatives ou un déficit auditif sont attendus chez près d'un tiers des enfants infectés pendant le premier trimestre. Certains de ces nouveau-nés sont encore asymptomatiques à la naissance et 10 à 15 % d'entre eux développeront des symptômes au cours des premières années de vie.<br>Bien que le risque de séquelles durables soit faible pour les fœtus infectés après le premier trimestre, les infections au deuxième et troisième trimestre peuvent causer une restriction de croissance fœtale et des contrôles de suivi sont alors recommandés.<br><br>La séroprévalence maternelle est d'environ 50 % en Europe occidentale. Le taux annuel de séroconversion chez les femmes enceintes dépend de la population et est compris entre 1 et 7 % dans les pays industrialisés ; en Europe occidentale, y compris la Suisse, on estime un taux de séroconversion plus faible d'environ 0,5 %. Rapporté au nombre de naissances vivantes en Suisse en 2014, cela correspondrait à 426 enfants avec une infection congénitale à CMV, dont près de 43 nouveau-nés avec une suspicion d'infection symptomatique à la naissance. Les statistiques hospitalières pour la Suisse font état de 23 infections symptomatiques pour cette période, mais le nombre de décès intra-utérins, fausses couches ou interruptions de grossesse n'est pas inclus dans ces données. L'infection maternelle se produit généralement par contact avec des sécrétions corporelles infectées (salive, urine, larmes, sécrétions génitales). La majorité des primo-infections maternelles à CMV sont asymptomatiques ; plus rarement des symptômes non spécifiques et grippaux apparaissent et les réinfections sont généralement asymptomatiques.	
**Populations à risque**<br>Un contact étroit avec de jeunes enfants de moins de 4 ans constitue le principal facteur de risque de séroconversion, les enfants infectés étant fréquemment excréteurs du virus pendant une période prolongée.<br>Les mères d'enfants en bas âge qui vont en crèche présentent un risque de séroconversion environ 10 fois supérieur.<br>Le risque de séroconversion au CMV est multiplié par 4 chez les puéricultrices (en crèche) par rapport au risque de base chez les femmes enceintes.	IIa
Selon les données disponibles, le personnel médical ne présente pas de risque accru de séroconversion au CMV par rapport au risque basal. Ce phénomène pourrait être dû aux mesures d'hygiène habituelles établies dans cet environnement.	III

**Evaluation du statut sérologique au CMV au début de la grossesse**<br>Toutes les femmes devraient être informées dans les plus brefs délais, idéalement avant la survenue de la grossesse, des enjeux de l'infection à CMV pendant la grossesse et de la possibilité de déterminer le statut sérologique au CMV. Ceci concerne particulièrement les femmes à risque accru d'exposition. La détermination du statut sérologique doit se limiter à la période préconceptionnelle et au premier trimestre, le risque de lésion fœtale étant considéré comme très faible au-delà de cette période (voir ci-dessus). L'existence d'une sérologie initiale préconceptionnelle peut aider à interpréter des résultats ambigus au début de la grossesse ou à planifier le moment d'une FIV en cas de détection d'une primo-infection.<br>En cas d'évaluation du statut sérologique au premier trimestre, l'examen devrait être effectué le plus tôt possible et inclure les IgG et les IgM anti-CMV. En cas de positivité des IgG et des IgM, l'avidité des IgG anti-CMV doit être mesurée pour estimer la date de l'infection (une faible avidité correspond à une infection récente, une avidité élevée à une infection plus ancienne). En cas de résultat négatif aux IgG et positif aux IgM, le test doit être répété après 2 semaines, les IgM pouvant être élevés de manière non spécifique (réaction croisée). En cas de positivité des IgG et de négativité des IgM, la patiente a déjà eu une infection à CMV par le passé. Mais elle n'est pas pour autant protégée contre une réinfection ou une réactivation. En cas de résultat négatif pour les IgG et les IgM, une seconde sérologie peut être réalisée à 12-14 semaines d'aménorrhée (SA) pour exclure une séroconversion dans l'intervalle. Au terme de cette période, l'évaluation du statut sérologique au moyen d'un dépistage n'est pas recommandée. La sérologie ne permet pas d'établir un diagnostic fiable de la réactivation ou de la réinfection.<br>Etant donné qu'il est difficile d'établir la date de la séroconversion, des résultats ambigus doivent être discutés avec un spécialiste en médecine fœto-maternelle pour guider la prise en charge ultérieure et éviter des interruptions de grossesse superflues.	
**Prévention par des mesures d'hygiène**<br>Il est fortement recommandé de lutter résolument contre les modes de transmission principaux par des mesures d'hygiène. Puisqu'un potentiel de risque existe aussi bien pour les primo-infections que pour les réinfections, la prévention touche toutes les femmes enceintes (y compris en cas de positivité des IgG anti-CMV), en particulier pendant la première moitié de la grossesse. La sensibilisation précoce des femmes enceintes aux risques spécifiques d'une infection au CMV et un conseil sur l'hygiène correspondante semblent réduire significativement le risque de séroconversion.<br>Lors de l'entretien indispensable sur les mesures préventives pendant la grossesse, le médecin traitant doit également aborder les risques d'une infection à CMV et le risque individuel de la femme enceinte (population à risque), car la prise de conscience est faible à ce sujet.<br>Les mesures d'hygiène suivantes sont recommandées : se laver soigneusement les mains à l'eau et au savon après tout contact avec des couches, de l'urine et des sécrétions corporelles de l'enfant comme la salive, les larmes et les sécrétions nasales. Eviter de partager les couverts et la vaisselle (nourriture et boissons communes), les brosses à dent, gants de toilettes et serviettes. Dans la mesure du possible, éviter d'embrasser les enfants en bas âge sur la bouche. Nettoyer les surfaces en contact avec la salive ou l'urine des enfants. Des gants à usage unique et des désinfectants pour les mains doivent également être mis à disposition dans les établissements où le risque de transmission du CMV est accru. Les recommandations d'hygiène s'adressent également au père de l'enfant qui, s'il est infecté, pourrait transmettre le virus à la femme enceinte par les sécrétions.	IIb
**Femmes présentant un risque accru d'exposition au CMV sur leur lieu de travail**<br>Toutes les femmes enceintes ou ne pouvant exclure une grossesse doivent être informées et averties par leur employeur des éventuels risques accrus d'infection à CMV sur leur lieu de travail, des conséquences possibles pour l'enfant à naître et de l'importance des mesures d'hygiène décrites ci-dessus, conformément à la loi (art. 63, al. 4 OLT 1). En plus des mesures d'hygiène, il faut éviter autant que possible le contact professionnel étroit entre les femmes enceintes et les enfants < 4 ans (changement de poste de travail), tout particulièrement jusqu'à 14 SA. Si cela n'est pas possible, les mesures d'hygiène doivent être strictement respectées et toutes les activités impliquant un contact potentiel avec des fluides corporels (changer la couche, nourrir, essuyer le nez/la bouche) doivent être réalisées avec des gants. Si les mesures d'hygiène ne peuvent pas être respectées pour des raisons opérationnelles et si l'affectation à un autre lieu de travail n'est pas possible, une interdiction d'occupation (inaptitude au travail selon l'ordonnance sur la protection de la maternité, https://www.seco.admin.ch/mutterschutz) devrait être prononcée, après une évaluation individuelle des risques, par le médecin traitant. Ces dispositions s'appliquent en particulier au premier trimestre. Une interdiction de travail généralisée n'est pas recommandée, pas plus qu'un arrêt maladie généralisé.	
**Conduite à tenir en cas de suspicion d'infection maternelle/fœtale à CMV**	

En cas de suspicion clinique ou d'échographie prénatale suspecte, il convient de procéder à un diagnostic CMV maternel. On commence par relever le statut sérologique complet (IgG/IgM anti-CMV). En cas de positivité des IgG et des IgM, l'avidité des IgG doit être déterminée afin de délimiter la date de l'infection (une faible avidité correspond à une infection récente, une avidité élevée à une infection plus ancienne), car la seule positivité des IgM a une faible valeur prédictive en ce qui concerne la primo-infection au CMV. La valeur pronostique d'une avidité élevée diminue toutefois à mesure que l'âge gestationnel avance. Si les résultats d'analyse indiquent une primo-infection maternelle ou une possible réinfection/réactivation ou s'ils sont ambigus, la patiente doit être adressée à un spécialiste en médecine fœto-maternelle pour des analyses complémentaires. Un examen invasif (PCR et charge virale dans le liquide amniotique ou, le cas échéant, prise de sang fœtal) doit être proposé et réalisé au plus tôt 8 semaines après la primo-infection présumée, idéalement après 18 à 20 SA. Un résultat d'amniocentèse positif au CMV avant 21 SA est diagnostique, mais une amniocentèse négative avant 21 SA (mais au plus tôt 8 semaines après l'infection) comporte un risque légèrement accru de résultats faux-négatifs, mais dans ces cas-là les nouveau-nés étaient toutefois asymptomatiques à la naissance. Une évaluation du risque lié à l'intervention par rapport aux conséquences du résultat (interruption de grossesse, traitement, participation à une étude) doit toujours précéder l'examen invasif.<br>En cas de détection d'une infection fœtale à CMV, la consultation et la prise en charge par un spécialiste en médecine fœto-maternelle sont recommandées afin d'évaluer les options thérapeutiques et de planifier un diagnostic échographique détaillé, une autre technique d'imagerie (IRM) et le monitorage fœtal.	
**Possibilités de traitement**	
En principe des agents antiviraux et de l'hyperimmunoglobuline spécifique au CMV peuvent être utilisés pour la prévention d'une transmission verticale en cas d'infection maternelle ou pour le traitement en cas d'infection fœtale à CMV établie. Des études très récentes, incluant un essai randomisé en double aveugle, contrôlé contre placebo, menées avec l'agent antiviral valaciclovir, révèlent une réduction significative des infections fœtales à CMV consécutives à une primo-infection maternelle acquise au début de la grossesse. Il existe également des données indiquant que le traitement par le valaciclovir peut, en cas d'infection fœtale à CMV confirmée, diminuer le risque d'infection symptomatique sous certaines conditions. Bien que ces études soient prometteuses et qu'aucun effet indésirable n'ait été décrit à ce jour, les expériences avec un traitement par le valaciclovir fortement dosé pendant la grossesse sont encore limitées et il convient d'évaluer soigneusement le rapport risque/bénéfice. Au cours de ces études le traitement prophylactique par le valaciclovir a été administré à raison de 8 g/d au total par voie orale (2x4 g/d ou 4x2 g/d), jusqu'au moment de l'amniocentèse. Des nouvelles données montrent qu'une dose de 4 x 2 g/d devrait être privilégiée, car le valaciclovir précipite dans les reins et peut, dans de rares cas, provoquer une insuffisance rénale aiguë. L'indication du traitement et la surveillance de la grossesse doivent être conduites par un spécialiste en médecine fœto-maternelle. Les contre-indications et les modalités de monitorage liées à l'administration de valaciclovir doivent être prises en considération. Le traitement est off-label, une demande de garantie de prise en charge doit être déposée auprès des caisses maladie.	Ib<br><br><br>IIb
En ce qui concerne l'hyperimmunoglobuline (HIG), deux études randomisées et contrôlées contre placebo n'ont montré aucun effet préventif significatif sur l'infection fœtale au CMV. Le rôle potentiel du moment de l'instauration du traitement, de l'intervalle d'administration et du dosage fait l'objet de discussions. Dans une des deux études un risque accru de naissance prématurée a été observé dans le groupe HIG, une constatation importante à prendre en considération. Une étude non randomisée, portant sur la prévention de la transmission par de l'hyperimmunoglobuline administrée à des intervalles raccourcis et intensifiés jusqu'à 20 SA, a révélé une diminution significative du taux de transmission intra-utérine lors de 40 primo-infections pendant le premier trimestre avec une instauration très rapide du traitement, comparé à un groupe témoin historique. La dose suivante a été administrée dans cette étude : HIG 200 IU/kg de poids corporel maternel une semaine sur deux jusqu'au moment de l'amniocentèse. Ce traitement pourrait être une bonne option en cas d'infection détectée très tôt D'autres ERC sont nécessaires avant de pouvoir émettre des recommandations à ce sujet. Le traitement étant off-label et très onéreux, une demande de garantie de prise en charge doit être déposée auprès des caisses maladie.	III
## Résumé<br>**L'infection congénitale à CMV est la cause la plus fréquente de malformations et de troubles du développement congénitaux d'origine infectieuse.**<br><br>**Un contact étroit avec des jeunes enfants < 4 ans constitue le principal facteur de risque de séroconversion maternelle.**	

Le risque de transmission verticale est relativement élevé et dépend de l'âge gestationnel. Selon les données actuelles, une transmission jusqu'à 12 à 14 SA expose le fœtus à un risque élevé de séquelles, alors que ce risque semble moindre lors d'une transmission plus tardive.

Toutes les femmes enceintes ou envisageant une grossesse doivent recevoir dès que possible des informations sur les risques d'infection à CMV et des recommandations portant sur les mesures d'hygiène préventives visant à diminuer ce risque. Par ailleurs elles devraient être informées de la possibilité d'évaluer le statut sérologique au CMV avant la conception et au début de la grossesse. Les avantages et désavantages d'avoir connaissance du statut sérologique doivent être discutés pour permettre une décision éclairée. Ceci doit également inclure le risque individuel d'infection à CMV de la femme (contact avec des enfants < 4 ans à la maison ou au travail, ou exposition présumée).

En cas (de suspicion) d'infection à CMV pendant la grossesse, la prise en charge devrait être assurée par un spécialiste en médecine fœto-maternelle. Selon des études récentes, il existe des options thérapeutiques efficaces pour réduire le risque de transmission verticale au début de la grossesse ainsi que pour améliorer l'issue d'un fœtus infecté.

Une interdiction de travail généralisée pour la femme enceinte à risque n'est pas recommandée et est uniquement indiquée si les mesures d'hygiène nécessaires ne peuvent pas être respectées pour des raisons opérationnelles. Ceci peut être limité au premier trimestre de la grossesse.

En dernier lieu il convient de procéder à une évaluation du nouveau-né en cas d'infection à CMV suspectée ou prouvée pendant la grossesse (confirmation de l'infection à CMV par PCR urinaire dans les 3 premières semaines de vie) par un néonatologue ou un pédiatre.

**Niveau de preuve**		**Grade de recommandation**	
**Ia**	Données probantes obtenues par la méta-analyse d'études randomisées et contrôlées	**A**	Dans la littérature, qui doit être globalement de bonne qualité et cohérente, il existe au moins une étude randomisée contrôlée ayant trait à la recommandation en question (niveaux de preuve Ia, Ib)
**Ib**	Données probantes obtenues à partir d'au moins une étude randomisée contrôlée	**B**	Le sujet de la recommandation est traité dans des études cliniques bien contrôlées mais qui ne sont pas randomisées (niveaux de preuve IIa, IIb, III)
**IIa**	Données probantes obtenues à partir d'au moins une étude contrôlée, bien menée, mais sans randomisation		On dispose de données probantes provenant de rapports ou d'avis de groupes d'experts ou de l'expérience clinique de spécialistes reconnus. Par contre, il n'existe pas d'études cliniques de bonne qualité qui soient directement applicables (niveau de preuve IV)
**IIb**	Données probantes obtenues à partir d'au moins une étude bien menée, d'un autre type, quasi expérimentale		**Le point de bonne pratique**<br>Traitement de choix, recommandé d'après l'expérience clinique du groupe d'experts ayant rédigé l'avis d'experts ou la directive
**III**	Données probantes obtenues à partir d'études descriptives, bien menées, non expérimentales, comme des études comparatives, des études de corrélation ou des études de cas		
**IV**	Données probantes obtenues à partir de rapports ou d'avis d'experts ou de l'expérience clinique de spécialistes reconnus		

Déclaration de conflits d'intérêts :
L'ensemble des auteurs déclarent n'avoir aucun conflit d'intérêts en rapport avec le présent avis d'experts.

Bibliographie : disponible auprès des auteurs

Date: 22 février 2021

La commission Assurance Qualité de gynécologie suisse / SGGO élabore des directives et des avis d'experts avec le plus grand soin ; toutefois la commission Assurance Qualité de gynécologie suisse / SGGO ne peut pas endosser la responsabilité de l'exactitude et de l'exhaustivité des contenus. Les informations des fabricants doivent être respectées dans tous les cas, en particulier les indications concernant la posologie.
Du point de vue de la commission, les directives et les avis d'experts correspondent à l'état actuel des connaissances scientifiques au moment de la rédaction. Les modifications survenues dans l'intervalle doivent être prises en compte par les utilisateurs.
